# CD8^+^ regulatory T cells shorten neutrophil lifespan to reduce hemorrhagic transformation after ischemic stroke

**DOI:** 10.1172/JCI203868

**Published:** 2026-09-01

**Authors:** Jianan Lu, Qia Zhang, Jiarui Chen, Huaming Li, An Ping, Ziyang Jin, Xiaotao Zhang, Yichen Gu, Xuejiao Dai, Zihong Chen, Yajun Qian, Guoqiang Zhang, Jun Yu, Jianmin Zhang, Ligen Shi

**Affiliations:** 1 Department of Neurosurgery, Second Affiliated Hospital, School of Medicine, Zhejiang University, Hangzhou, Zhejiang, China.; 2 Clinical Research Center for Neurological Diseases of Zhejiang Province, Hangzhou, Zhejiang, China.; 3 State Key Laboratory of Transvascular Implantation Devices, Hangzhou, Zhejiang, China.; 4 Department of Health Management Center, Second Affiliated Hospital, School of Medicine, Zhejiang University, Hangzhou, Zhejiang, China.; 5 Brain Research Institute, Zhejiang University, Hangzhou, Zhejiang, China.; 6 Research Center for Life Science and Human Health, Binjiang Institute of Zhejiang University, Hangzhou, Zhejiang, China.

**Keywords:** Immunology, Neuroscience, Vascular biology, Neutrophils, Stroke, T cells

## Abstract

The neutrophil-to-lymphocyte ratio (NLR) is associated with unfavorable prognosis and hemorrhagic transformation (HT) in patients with ischemic stroke, yet the underlying mechanisms remain unclear. Using patient samples and a murine stroke model, we identified CD8^+^ regulatory T cells (CD8 Tregs) as key regulators of neutrophil homeostasis after ischemic stroke, thereby limiting endothelial disruption and HT. Loss of CD8 Tregs expanded circulating neutrophils by extending their lifespan rather than altering proliferation, bone marrow release, or direct cytotoxicity. Mechanistically, CD8 Tregs shortened neutrophil lifespan by modulating HIF-1α–dependent glycolytic activity and relieving PD-L1–mediated suppression of bone marrow clearance. Finally, coculture experiments with human CD8 Tregs and neutrophils revealed similar neutrophil-regulatory effects, accompanied by improved endothelial barrier integrity. These findings reveal a previously unrecognized CD8 Treg–neutrophil axis and suggest potential therapeutic strategies for preventing HT after stroke.

## Abstract 2

CD8 regulatory T cells protect against brain bleeding after ischemic stroke by shortening neutrophil lifespan and limiting their harmful effects.

## Graphical abstract

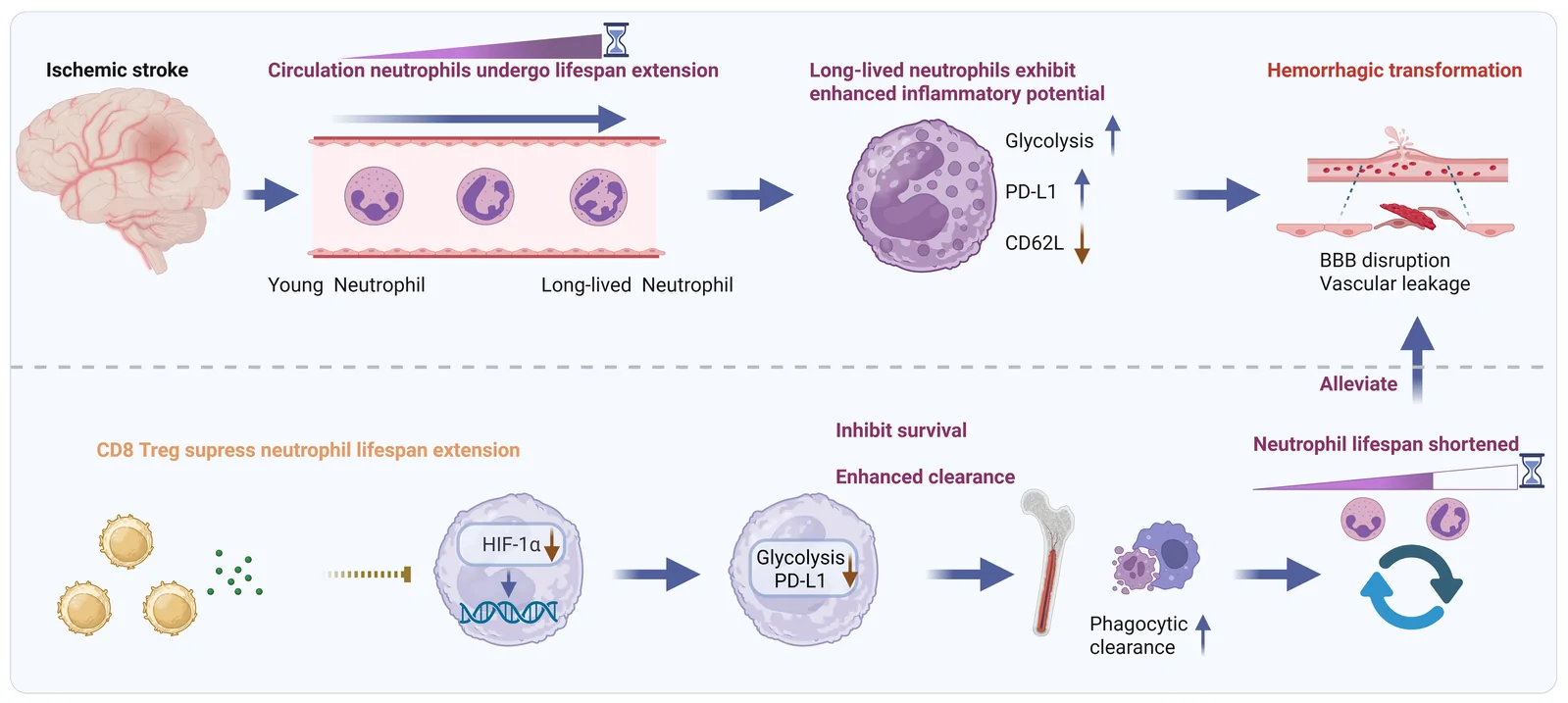

## Introduction

Hemorrhagic transformation (HT) is a common complication of ischemic stroke that arises from endothelial barrier disruption, with its incidence further increased in patients undergoing reperfusion therapies (1–4). HT is closely associated with unfavorable neurological recovery and, when severe, markedly increased mortality (2–4). Although several clinical indicators have been proposed as potential predictors of HT (5), effective strategies for its prevention and targeted intervention remain lacking.

The neutrophil-to-lymphocyte ratio (NLR) has been extensively investigated in ischemic stroke, where elevated NLR is consistently linked to both the occurrence of HT and adverse outcomes (6–8). However, the mechanisms underlying alterations in NLR during HT remain poorly understood. It is unclear whether these changes merely accompany disease progression or instead represent a driving factor. Given its mathematical definition, NLR increases when circulating neutrophils rise relative to lymphocytes, suggesting that it may also serve as an indicator of peripheral immune imbalance.

It is well established that peripheral immune cells play a critical role in poststroke pathology and the development of HT (9–11). As the principal contributors to NLR, neutrophils respond rapidly after stroke and promote the disruption of blood–brain barrier (BBB) integrity by releasing multiple proinflammatory mediators, such as matrix metalloproteinase-9 (MMP-9) and neutrophil extracellular traps (NETs), thereby inducing and exacerbating HT (12–14). Importantly, accumulating evidence highlights that neutrophils are not a homogeneous population but rather exhibit substantial heterogeneity (15). In peripheral circulation, neutrophils exist in different maturation states, with long-lived senescent or terminally differentiated cells showing heightened proinflammatory activity and greater vascular-damaging potential (16–18). However, little is currently known about how neutrophil lifespan dynamics influence changes in NLR and the occurrence of HT.

On the other hand, both T cells and B cells have been reported to regulate neutrophil function in various disease contexts (19–21). The protective role of T cells in preventing HT indicates that lymphopenia may serve as a risk factor (22), implying that lymphocytes may participate in poststroke pathological processes from a different perspective. Moreover, previous studies have suggested that T cell death may modulate neutrophil lifespan (23). Nevertheless, the functional properties of lymphocyte subsets and their regulatory mechanisms vary considerably with the disease environment (19, 24–26). In addition, whether lymphocyte-mediated regulation of neutrophil lifespan contributes to changes in NLR during HT remains unclear.

Therefore, elucidating the mechanisms underlying NLR alterations during the development of HT, and determining whether these changes reflect impaired lymphocyte-mediated regulation of neutrophils, is of great importance for identifying potential therapeutic targets. In this study, analysis of blood samples from patients who have had an ischemic stroke and murine stroke models revealed that elevated NLR in those with HT was associated with defective T cell regulation of neutrophils. Moreover, we identified CD8^+^ regulatory T cells (CD8 Tregs) as key endogenous controllers of neutrophil lifespan, acting through HIF-1α–mediated metabolic regulation and modulation of neutrophil clearance in the bone marrow. Finally, this regulatory process was validated by coculture of CD8 Tregs and neutrophils isolated from patient blood. Collectively, these findings provide new insights into immunotherapeutic strategies for HT and ischemic stroke.

## Results

### NLR reflects T cell–mediated regulation of neutrophils in predicting prognosis after ischemic stroke.

We conducted a cohort study involving patients with acute ischemic stroke admitted to our center. Peripheral blood samples were collected at admission to characterize immune profiles during the acute phase (Figure 1A). Based on the occurrence of HT after stroke onset, patients were classified into HT and non-HT (NHT) groups (Supplemental Figure 1A and Supplemental Table 1; supplemental material available online with this article; https://doi.org/10.1172/JCI203868DS1). Functional outcomes were assessed using the modified Rankin Scale (mRS) at 3 months to evaluate differences in recovery between the 2 groups (Figure 1A). We observed that patients in the HT group had a markedly higher NLR and worse functional recovery, as reflected by higher mRS scores at 3 months (Figure 1, B and C). Moreover, elevated NLR correlated with poorer functional recovery at both discharge and 3 months and showed stronger predictive ability for severe unfavorable outcomes, defined as an mRS score ≥ 5 (Supplemental Figure 1, B–E). We further quantified hemorrhage volume in patients with HT who developed substantial parenchymal hematomas and found that NLR was positively correlated with hemorrhage volume (Figure 1D). Collectively, these clinical findings indicate that NLR is strongly associated with both severe HT and unfavorable functional outcomes after stroke, consistent with previous reports (6–8).

To investigate the underlying causes of NLR alterations, we employed a murine model of ischemic stroke induced by middle cerebral artery occlusion (MCAO). Mice were stratified into HT and NHT groups at day 3 after MCAO, based on the presence or absence of visible hemorrhagic foci or hematoma on the brain surface or within the ischemic region upon tissue harvest (Supplemental Figure 1F). Consistent with the elevated NLR in patients, the ratios of neutrophils to both T lymphocytes and B lymphocytes were markedly increased in the HT group (Figure 1, E and F, and Supplemental Figure 1G). This increase was mainly driven by elevated peripheral neutrophil numbers, as T and B cell numbers remained largely unchanged (Supplemental Figure 1H).

To dissect alterations in the peripheral immune landscape, we conducted single-cell RNA sequencing (scRNA-seq) of peripheral blood immune cells from HT and NHT mice after MCAO. Following quality control, 12,052 cells were clustered into 6 immune cell populations: B cells, neutrophils, CD8 T cells, monocytes, CD4^+^ T cells (CD4 T cells), and NK cells (Figure 1G and Supplemental Figure 2A). Quantitatively, the proportion of neutrophils was markedly increased in the HT group, whereas the proportions of B cells and CD8 T cells were significantly decreased, with CD4 T cells showing a slight increase (Figure 1G). Functional analysis revealed that neutrophils in the HT group exhibited more pronounced activation and increased cytokine production, whereas B cells, CD4 T cells, and CD8 T cells did not display evident proinflammatory phenotypic changes, highlighting neutrophils as a prominent proinflammatory population in HT (Supplemental Figure 2, B–E). In addition, HT mice also exhibited more severe disruption of endothelial barrier integrity and increased neutrophil infiltration within the infarcted brain regions (Figure 1, H and I). To validate these findings in a clinical context, bulk RNA-seq of peripheral blood neutrophils from patients with HT and NHT revealed distinct transcriptional states, with HT neutrophils upregulating proinflammatory pathways and genes implicated in vascular damage, including *CD177*, *MMP9*, and *IL1R1* (Figure 1J, and Supplemental Figure 2F). Together, these findings indicate that peripheral neutrophils act as key executors in the initiation and progression of HT, driven by their increased abundance and heightened proinflammatory effector activity.

To investigate the potential regulatory role of lymphocytes on neutrophils in the context of HT, we employed *Rag1^–/–^* mice, which lack mature lymphocytes, and established an MCAO model (Figure 1K). We found that at both day 1 and day 3 after MCAO, *Rag1^–/–^* mice exhibited significantly increased peripheral neutrophil counts (Figure 1L). Correspondingly, increased neutrophil infiltration was also detected in the brains of *Rag1^–/–^* mice on day 3 (Supplemental Figure 2, G and H). Moreover, this trend of elevated neutrophil abundance in both the blood and the brain persisted into the subacute phase at day 7 (Figure 1L and Supplemental Figure 2H). To determine whether and which type of lymphocyte depletion affects peripheral neutrophil numbers, we adoptively transferred either T cells or B cells into *Rag1^–/–^* mice (Figure 1M). The results showed that T cell transfer significantly reduced neutrophil counts, whereas B cell transfer had no effect (Figure 1N). In addition, scRNA-seq data revealed that T cells exhibited stronger interaction networks with neutrophils compared with B cells (Figure 1O and Supplemental Figure 2I). These findings suggest that elevated NLR reflects a peripheral immune imbalance between T cells and neutrophils following stroke, which is associated with increased risk of HT and unfavorable outcomes.

To further explore the relationship between lymphocytes and neutrophils within the brain after stroke, we performed scRNA-seq analysis of immune cells isolated from the brains of HT and NHT mice. After quality control, a total of 10,062 cells were clustered into distinct immune populations, including macrophages, neutrophils, dendritic cells, T cells, and microglia, based on marker expression (Supplemental Figure 3, A and C). Among these populations, neutrophils and macrophages constituted the major infiltrating immune cells during the acute phase after stroke (Supplemental Figure 3B). Notably, neutrophils were present at a substantially higher proportion in the brains of HT mice compared with NHT mice and were characterized by enhanced immune effector features, including increased expression of genes associated with adhesion, chemotaxis, and inflammatory cytokine responses (Supplemental Figure 3, B and D). In contrast, T cell infiltration into the brain remained relatively limited during the acute phase (Supplemental Figure 3B), suggesting that the markedly elevated neutrophil infiltration and inflammatory activity observed in the brains of HT mice are more likely regulated by peripheral T cells rather than by T cells acting directly within the CNS parenchyma.

### Peripheral CD8 Tregs act as endogenous regulators of neutrophils after stroke.

To further explore how T cells regulate neutrophils, we analyzed peripheral T cell subsets in the HT and NHT groups. Based on the expression of surface markers *Cd44* and *Sell (encoding* CD62L*)*, T cells were classified into CD4 naive, CD8 naive, and effector/memory populations with functional or memory characteristics (CD4 effector/memory [CD4 E/M] and CD8 E/M), as well as IFN-responsive T cells (IFN-T) (Figure 2A and Supplemental Figure 4A). Functionally, among T cell subsets, CD8 E/M and CD4 E/M cells exhibit stronger immunoregulatory capacity in restraining excessive immune responses compared with other T cell populations (Figure 2B). Among T cell populations reduced in HT mice, both CD8 naive and CD8 E/M were decreased, with the decline in CD8 E/M reaching approximately 50% (Figure 2A and Supplemental Figure 4B). We then examined surface molecules preferentially expressed on CD8 E/M cells and identified *Il2rb* as being predominantly expressed in this subset (Figure 2C and Supplemental Table 2). Notably, analysis of samples from patients who had had strokes further revealed a significantly lower proportion of CD122^hi^ T cells (encoded by *Il2rb*) in the HT group compared with the NHT group (Figure 2D and Supplemental Figure 4C).

We then speculated that depletion of CD122^hi^ T cells might alter neutrophil abundance after stroke. To test this, we administered a CD122 antibody (CD122Ab) in vivo, which selectively targets CD122-expressing cells (Figure 2E). This intervention resulted in a marked reduction in the proportion of CD44^+^ CD8 E/M cells compared with the isotype control group (Supplemental Figure 4D). By contrast, the proportions of CD4 naive, CD8 naive, and CD4 E/M cells remained unchanged or slightly increased, consistent with their relatively low expression of *Il2rb* (Supplemental Figure 4, A, E, and F). Most importantly, on day 3 after MCAO, both peripheral and brain-infiltrating neutrophil numbers were significantly elevated in the CD122Ab-treated group (Figure 2, F and G). Furthermore, we found that the increase in neutrophil abundance was accompanied by more severe HT, pronounced endothelial barrier disruption, and increased leakage of endogenous IgG into the brain in CD122Ab-treated mice at day 3 after MCAO, indicative of aggravated BBB damage (Figure 2, H and I, and Supplemental Figure 5A).

To investigate whether CD122Ab might exert off-target effects on the overall peripheral immune landscape, we performed scRNA-seq on peripheral immune cells from CD122Ab-treated and control mice. After quality control, a total of 21,734 cells were subjected to downstream analyses (Figure 2J). In addition to the validated reduction of CD8 E/M populations, we also observed decreases in NK and B cell numbers, whereas no significant changes were detected in other immune cell populations (Figure 2J). Given that *Il2rb* is highly expressed on NK cells but only minimally expressed on B cells (Supplemental Figure 5B), the reduction in B cells is more likely an indirect effect secondary to CD122^hi^ cell depletion. A prior study also reported that NK cells express high levels of CD122 (27), and consistent with this, CD122Ab administration markedly reduced NK cell numbers in MCAO mice (Supplemental Figure 5C). To determine whether the effect of CD122Ab on neutrophil abundance is mediated through NK cells, we directly depleted NK cells by administering NK1.1Ab in *Rag1^–/–^* mice and found no significant changes in peripheral neutrophil numbers at day 3 after stroke (Supplemental Figure 5D). Furthermore, administration of CD122Ab in *Rag1^–/–^* mice likewise produced no significant changes in peripheral or brain-infiltrating neutrophil numbers (Supplemental Figure 5E). These findings suggest that CD122Ab regulates neutrophil abundance through CD122^hi^ T cells, rather than through NK cell–mediated or T cell–independent direct effects on neutrophils. In addition, given that CD4 T cells, including CD4 Tregs, have also been reported to regulate neutrophil abundance and function (28, 29), we further examined the effects of CD122Ab on these populations using *Foxp3* reporter mice (Supplemental Figure 5F). No significant changes were observed in either total CD4 T cell or CD4 Treg numbers following CD122Ab treatment (Supplemental Figure 5, G and H). Taken together, these findings indicate that CD122^hi^ T cells are the primary mediators of the neutrophil changes observed following CD122Ab treatment.

We next profiled CD122^hi^ T cells, which predominantly comprise CD8 E/M subsets, to determine which populations were affected by CD122Ab treatment. Unsupervised clustering based on differential marker expression classified these cells into CD8 memory subsets and several additional functional subsets, including CD8 effector T cells (CD8 EF), natural killer T (NKT) cells, γδ T cells, and a unique CD8 Treg subset characterized by high *Ly49* gene expression (Supplemental Figure 5, I and J). Notably, the proportions of CD8 EF, NKT cells, and CD8 Tregs were reduced following CD122Ab treatment (Figure 2K). Nevertheless, CD8 EF, NKT cells, and CD8 Tregs displayed distinct gene expression signatures (Supplemental Figure 5J). For example, in addition to *Ly49*, CD8 Tregs expressed *Ikzf2* (Supplemental Figure 5J), which has been reported to be critical for their immunoregulatory function (30). Thus, to examine the relationship between these cell populations and neutrophil counts, we adoptively transferred each of the 3 subsets into mice (Figure 2L, Supplemental Figure 5K). Neutrophil numbers were decreased only in mice receiving CD8 Tregs, whereas transfer of CD8 EF or NKT cells had no significant effect (Figure 2M). Correspondingly, adoptive transfer of CD8 Tregs, but not CD8 EF or NKT cells, alleviated HT after MCAO (Figure 2N). Moreover, CD8 Treg transfer attenuated BBB disruption and reduced endogenous IgG leakage, indicating improved endothelial barrier integrity (Supplemental Figure 5, L and M). Collectively, these findings indicate that CD8 Tregs act as endogenous regulators of peripheral neutrophils.

### CD8 Tregs are rapidly mobilized after stroke but undergo enhanced apoptosis in HT mice.

To investigate the dynamic response of CD8 Tregs following stroke, we examined changes in CD8 Treg numbers at day 1 after MCAO, a hyperacute time point at which CD8 Tregs have been reported to represent among the earliest lymphocyte populations to respond to stroke (31). Flow cytometric analysis demonstrated a significantly higher proportion of CD8 Tregs in MCAO mice compared with sham controls at day 1 after MCAO (Figure 3A), suggesting early activation and mobilization of CD8 Tregs following ischemic injury. To further characterize the transcriptional basis of this early response, we performed bulk RNA-seq of peripheral CD8 Tregs isolated at day 1 after MCAO. The results revealed notable transcriptional changes at this early time point, characterized by upregulation of gene signatures associated with T cell activation, immunoregulatory function, cytokine production, migration, and TCR signaling (Figure 3B and Supplemental Figure 6A). Flow cytometric analysis of previously reported key effector molecules and transcription factors of CD8 Tregs (30, 32, 33) revealed that Helios expression was significantly elevated in MCAO CD8 Tregs compared with sham controls, whereas Eomes expression was not significantly altered (Figure 3C and Supplemental Figure 6B). In contrast, Perforin expression was significantly downregulated following MCAO (Supplemental Figure 6B), indicating a reduction in direct cytotoxic capacity. These findings suggest that CD8 Tregs undergo rapid mobilization and activation within the hyperacute phase after stroke, a response pattern that may differ substantially from the kinetics of other T cell populations.

To further explore alterations in CD8 Tregs during HT, we analyzed CD8 Treg changes in HT and NHT mice at day 3 after MCAO. scRNA-seq analysis revealed a lower proportion of CD8 Tregs within peripheral blood T cells in HT mice compared with NHT mice (Figure 3D and Supplemental Figure 6C), a finding confirmed by flow cytometric analysis (Figure 3E). Notably, baseline CD8 Treg numbers prior to MCAO induction were broadly comparable between mice subsequently classified as HT and NHT (Supplemental Figure 6D), suggesting that preexisting interindividual differences in CD8 Treg abundance are unlikely to account for the differential outcomes between groups. In contrast with the marked reduction in CD8 Treg abundance, differential gene expression analysis revealed no substantial transcriptional differences between HT and NHT CD8 Tregs (Supplemental Figure 6E). Of note, genes related to cellular activation, immune suppression, lineage stability, migration, and metabolic programming did not differ significantly between groups (Supplemental Figure 6F). Flow cytometric analysis further showed that expression levels of key effector molecules and transcription factors were comparable between HT and NHT CD8 Tregs (Supplemental Figure 6G). These results suggest that a decrease in CD8 Treg abundance, rather than overt alterations in their functional state, may be more closely associated with the development of HT.

We next explored the potential mechanisms underlying this decrease. Given that bulk RNA-seq of MCAO CD8 Tregs at day 1 revealed upregulation of apoptosis-related pathways and suppression of metabolic activity (Figure 3B), we first examined CD8 Treg apoptosis at day 3 after MCAO. The proportion of apoptotic peripheral CD8 Tregs was significantly increased in HT mice compared with NHT mice (Figure 3F). In addition, brain infiltration of CD8 Tregs at day 3 after MCAO did not differ between HT and NHT mice, with the absolute numbers of infiltrating CD8 Tregs remaining markedly limited in both groups (Supplemental Figure 6H). These findings suggest that the decline in CD8 Tregs in HT mice is primarily associated with enhanced peripheral apoptosis rather than increased brain infiltration. To clarify the temporal relationship between CD8 Treg apoptosis and HT development, we quantified apoptotic CD8 Tregs at day 1 after MCAO and found that HT mice already exhibited a significantly higher proportion of apoptotic CD8 Tregs at this early time point (Supplemental Figure 6I). The early onset of CD8 Treg apoptosis suggests that CD8 Treg loss may contribute to HT susceptibility.

### CD8 Tregs shorten neutrophil lifespan to reduce their numbers in circulation after ischemic stroke.

We next explored the mechanisms by which CD8 Tregs regulate neutrophils. scRNA-seq analysis revealed functional signatures in CD8 Tregs related to the negative regulation of cell activation and the promotion of cell death (Supplemental Figure 7A). To functionally validate these signatures, we cocultured CD8 Tregs with neutrophils and found that CD8 Tregs suppressed proinflammatory factor expression while promoting antiinflammatory factor expression in neutrophils (Figure 3G), suggesting that CD8 Tregs can modulate the functional states of neutrophils.

We then examined whether CD8 Tregs directly induce neutrophil death. To address this, CD8 Tregs were adoptively transferred into MCAO mice, and peripheral neutrophil apoptosis was subsequently assessed. Notably, neutrophil apoptosis was comparable between CD8 Treg–treated and control mice (Figure 3H). We further performed 6-hour in vitro coculture assays, corresponding to the midpoint of the previously reported neutrophil half life (32). Consistent with the in vivo findings, CD8 Treg coculture did not significantly alter neutrophil apoptosis (Figure 3I), suggesting that the reduction in neutrophil numbers is unlikely to result from direct apoptosis induction by CD8 Tregs.

We next examined whether the increase in neutrophil numbers following CD8 Treg depletion was attributable to enhanced bone marrow production or release. BrdU was administered prior to MCAO, thereby serving as a proliferation label for cells generated after MCAO (Figure 3J). Analysis of femoral bone marrow showed that approximately 70%–80% of neutrophils were BrdU^pos^, representing newly generated neutrophils after MCAO and indicating a rapid bone marrow response to injury (Supplemental Figure 7B). However, no significant difference in neutrophil proliferation was observed between the isotype control and CD122Ab treated groups (Supplemental Figure 7B). In addition, because myeloid cells generated within the skull bone marrow have been reported to directly infiltrate the brain after injury (34, 35), we also analyzed neutrophil proliferation within the calvarial bone marrow. Similarly, CD122Ab treatment did not significantly alter neutrophil proliferation in the calvarial bone marrow compartment (Supplemental Figure 7C). Interestingly, peripheral blood results revealed that, in CD122Ab-treated mice, the proportion of BrdU^neg^ neutrophils representing neutrophils present prior to MCAO was significantly increased, pointing to a possible prolongation of neutrophil survival (Figure 3K). To test this directly, we transferred neutrophils isolated from CD45.1 donor mice into isotype or CD122Ab treated WT recipients and found that circulating neutrophils persisted longer in CD122Ab-treated mice compared with isotype controls (Figure 3, L and M).

To further investigate the basis for altered neutrophil lifespan, we analyzed neutrophil subpopulations and identified 4 clusters (Figure 4A, Supplemental Figure 7D). Comparison of these clusters with previously reported blood neutrophil subsets revealed that Neu1 corresponded to G3 and G4 described by Xie et al. (16) and to scNeu1 reported by Gullotta et al. (36); Neu2 resembled G5a; Neu3 was similar to scNeu2/4; and Neu4 corresponded to G5c and scNeu3 (Figure 4A). Notably, the transition from G3/4 to G5c, or from scNeu1 to scNeu4, has been proposed to represent the progression of neutrophils from a mature state to a terminal/senescent state (16, 36). In our dataset, trajectory analysis performed with scTour, Monocle3, and CytoTRACE2 revealed continuous progression along neutrophil states, with Neu3 and Neu4 positioned at the terminal end of the trajectory (Figure 4B and Supplemental Figure 7E). In addition, we observed differences in neutrophil subset composition between the groups, characterized by an increased proportion of the terminal-state populations Neu3 and Neu4 after CD122Ab treatment (Figure 4C and Supplemental Figure 7F). Monocle2 was used to construct a pseudotime trajectory of neutrophils, revealing a shift in state progression between the 2 groups, with CD122Ab treated cells distributed toward the late pseudotime (Figure 4D). We further evaluated nuclear morphology by subjecting peripheral neutrophils from both groups to Giemsa staining, which demonstrated a significantly higher proportion of hypersegmented nuclei in the CD122Ab group, providing additional evidence for an overall prolongation of neutrophil lifespan (Figure 4E).

We next examined transcriptional signatures associated with terminal neutrophil states. The G5c signature progressively increased from Neu1 to Neu4, with CD122Ab treatment further increasing G5c scores across neutrophil subsets (Figure 4F). Consistently, the neutrophil aging score (16) progressively increased from Neu1 to Neu4 and was higher in CD122Ab-treated mice than in isotype controls (Figure 4F and Supplemental Figure 7G). To further validate this transcriptional shift, we performed bulk RNA-seq on peripheral neutrophils from isotype- and CD122Ab-treated MCAO mice at day 3. CD122Ab treatment increased the expression of late neutrotime genes, representing transcriptional programs associated with the terminal stage of neutrophil maturation (37) (Figure 4G). Together, these findings suggest that CD122Ab induces a global shift in neutrophils toward a prolonged-lifespan phenotype, which we refer to as the long-lived state.

CD62L downregulation is characteristic of neutrophils at later stages of their lifespan (17, 18). We next adoptively transferred CD8 Tregs into MCAO mice and found significantly increased CD62L expression in both peripheral blood and brain-infiltrating neutrophils at day 3 after MCAO, suggesting that CD8 Tregs suppress neutrophil lifespan extension (Figure 4, H–J). This effect was further supported by in vitro coculture experiments, in which CD8 Tregs, but not other CD8 T cells, significantly increased CD62L expression on neutrophils (Figure 4K). Since Qa-1 (encoded by *H2-T23*) has been reported to serve as an important mediator of CD8 Treg function (38, 39), we examined its expression in neutrophils and found increased Qa-1 expression following CD122Ab treatment (Supplemental Figure 7H). Moreover, Qa-1 expression was predominantly enriched in the long-lived neutrophil subset in CD122Ab-treated mice, suggesting that Qa-1 expression on neutrophils may be regulated by CD8 Tregs (Supplemental Figure 7I). Given that Qa-1–dependent CD8 Treg activity has been reported to involve direct cell-cell contact with target cells (40), we further performed direct and indirect (transwell) coculture assays between CD8 Tregs and neutrophils. Notably, CD62L expression remained comparable between the 2 conditions (Supplemental Figure 7J), suggesting that, although neutrophil Qa-1 expression may be influenced by CD8 Tregs, the regulation of neutrophil lifespan by CD8 Tregs is unlikely to depend primarily on Qa-1–mediated direct cell-cell interactions.

Previous studies have suggested that long-lived or senescent neutrophils are linked to enhanced inflammatory function (17, 18, 41, 42). Moreover, scRNA-seq data revealed functional distinctions between early-stage and long-lived neutrophils, with long-lived neutrophils exhibiting stronger capacities for cell adhesion, antigen presentation, cytokine production, and BBB disruption (Figure 4L). Functional enrichment analysis of our bulk RNA-seq data also demonstrated that neutrophils from CD122Ab-treated MCAO mice exhibited significant upregulation of proinflammatory functions, including cytokine production and inflammatory response pathways, following stroke (Supplemental Figure 7K). To test whether CD8 Tregs limit neutrophil-mediated BBB disruption, we employed an in vitro endothelial cell oxygen-glucose deprivation (OGD) model to mimic the environment after a stroke (Figure 4M). Coculture with CD8 Tregs suppressed neutrophil transendothelial migration under both physiological and LPS-stimulated conditions (Figure 4N). In addition, CD8 Treg coculture attenuated neutrophil-mediated disruption of endothelial integrity (Figure 4O and Supplemental Figure 7L), whereas coculture of CD8 Tregs alone with OGD-pretreated endothelial cells had no significant effect, suggesting that neutrophils serve as intermediaries mediating the endothelial-protective function of CD8 Tregs (Figure 4O). To further validate this, we performed MCAO in neutrophil-depleted mice with adoptive transfer of CD8 Tregs. In this setting, endogenous IgG leakage was not significantly altered, indicating that CD8 Tregs limit BBB disruption after stroke through regulation of neutrophils (Figure 4P).

Collectively, these results suggest that CD8 Tregs regulate neutrophils primarily by shortening their lifespan, thereby constraining neutrophil abundance and inflammatory activity within a controlled range.

### CD8 Tregs limit neutrophil lifespan by suppressing glycolysis and alleviating PD-L1–mediated inhibition of phagocytic clearance.

To further elucidate the mechanisms by which CD8 Tregs shorten neutrophil lifespan, we performed transcriptomic profiling of neutrophils cultured in vitro for different durations (Figure 5A). PCA revealed that freshly isolated neutrophils (0 h) and those cultured for 24 or 48 hours exhibited distinct transcriptomic signatures (Supplemental Figure 8A). It is worth noting that neutrophils cultured for 24 or 48 hours showed heightened inflammatory responses compared with freshly isolated cells (Supplemental Figure 8, B and C), in line with our scRNA-seq observation that long-lived neutrophils in vivo exhibit enhanced inflammatory functions.

To construct a gene expression model associated with lifespan extension, we next clustered genes according to their temporal expression dynamics during prolonged culture. The clustering approach was designed to assign each gene to a single cluster, thereby enhancing the distinctiveness of cluster membership. Two clusters exhibited higher expression at 48 hours than at earlier time points: cluster A genes increased progressively over time, whereas cluster B genes remained largely unchanged at 24 hours but rose sharply with extended culture (Figure 5B and Supplemental Table 3). Integration of these signatures with the scRNA-seq dataset revealed that cluster A expression increased gradually from Neu1 to Neu4, whereas cluster B expression was more pronounced specifically in Neu4 (Figure 5B). We then compared the expression of these 2 gene signatures between the isotype and CD122Ab groups and observed significant upregulation of both cluster A and cluster B genes in neutrophils of the CD122Ab group (Figure 5C). These results indicate that the in vitro model recapitulates the transcriptional dynamics associated with in vivo neutrophil lifespan progression.

Given that the loss of CD8 Tregs induced by CD122Ab was identified as the cause of neutrophil lifespan extension, we next employed an in vitro coculture model followed by RNA-seq to identify lifespan-associated genes subject to CD8 Treg regulation (Figure 5D). Transcriptomic analysis of neutrophils cocultured with CD8 Tregs revealed that a substantial proportion of downregulated genes were associated with proinflammatory functions (Supplemental Figure 8, D–F). In addition, the expression of several genes linked to the long-lived neutrophil state was also suppressed (Supplemental Figure 8G). By intersecting the genes upregulated in neutrophils cultured without CD8 Tregs with those in clusters A and B, we identified a core set of long-lived state–associated genes that could be downregulated by CD8 Tregs (Figure 5D). Among these, genes intersecting with cluster A were enriched in pathways related to inflammatory responses, cytokine production, apoptosis, cell adhesion, and signaling cascades such as the PD-L1 and TLR2 pathways. In parallel, genes intersecting with cluster B were associated with autophagy, chemotaxis, secretion, metabolism, and the HIF signaling pathway (Figure 5D).

It is well established that neutrophil survival and the execution of their immune functions depend on energy acquisition (43, 44). Accordingly, we noted that the expression of *Slc2a1*, as well as its encoded protein GLUT1, was markedly increased at 48 hours compared with 0 hours, a pattern consistent with the cluster B gene signature, whereas coculture with CD8 Tregs significantly suppressed their expression in neutrophils (Supplemental Figure 8, H–J). Given that GLUT1 is a key glucose transporter in neutrophils (45), these findings raise the possibility that CD8 Tregs may regulate neutrophil lifespan, at least in part, by modulating cellular metabolism. We therefore performed metabolic profiling of neutrophils following in vitro coculture. Compared with neutrophils cultured alone, coculture with CD8 Tregs markedly reduced glycolytic activity, whereas amino acid metabolic products were elevated in the single-culture group (Figure 5E and Supplemental Figure 8K). In addition, we examined the extracellular acidification rate (ECAR) in the coculture model and found that CD8 Tregs significantly suppressed neutrophil glycolysis. Of note, although glycolytic reserve was slightly reduced in the coculture compared with the single-culture condition, it remained minimal, indicating that glycolysis is the predominant energy source for neutrophils (Figure 5F and Supplemental Figure 8L).

We next inhibited glycolysis in vivo using 2-deoxy-D-glucose (2-DG) and assessed whether this intervention altered CD8 Treg-mediated regulation of neutrophil lifespan as well as HT and BBB injury after MCAO. Consistent with previous reports (46), glycolysis inhibition significantly improved neurological recovery after MCAO (Supplemental Figure 9, A and B). In addition, inhibition of glycolysis significantly reduced circulating neutrophil numbers and the proportion of hypersegmented neutrophils, accompanied by markedly increased CD62L expression, suggesting that glycolysis contributes to neutrophil lifespan extension after stroke (Figure 5, G and H, and Supplemental Figure 9, C–E). Furthermore, circulating neutrophil abundance, the proportion of hypersegmented neutrophils, and CD62L expression were not additionally affected by CD8 Treg transfer under glycolysis-inhibited conditions (Figure 5, G and H, and Supplemental Figure 9, C–E). We further examined the effects of glycolytic inhibition on HT and BBB integrity after MCAO. Notably, glycolytic inhibition significantly alleviated HT and attenuated BBB disruption following stroke. However, under these conditions, adoptive transfer of CD8 Tregs conferred no additional protective effects (Supplemental Figure 9, F and G). Together, these findings suggest that CD8 Tregs regulate neutrophil lifespan and exert protective effects against HT at least partly through suppression of neutrophil glycolysis.

Among the core genes associated with the long-lived neutrophil state regulated by CD8 Tregs, we also noted that *Cd274* (encoding PD-L1) expression progressively increased with culture duration, consistent with the cluster A expression pattern (Figure 5I). Consistently, PD-L1 protein levels were significantly increased after 48 hours of culture (Figure 5J). Given previous reports that PD-L1 expression can extend neutrophil survival (47), we hypothesized that PD-L1 may mediate the regulation of neutrophil lifespan by CD8 Tregs. Consistent with this hypothesis, scRNA-seq analysis revealed significantly elevated *Cd274* expression in neutrophils from CD122Ab-treated mice compared with isotype controls (Figure 5K). Notably, this upregulation was concentrated in terminal long-lived neutrophil subsets, whereas expression in the corresponding populations of the isotype group was minimal (Figure 5L). Flow cytometric analysis of peripheral blood at day 3 after MCAO revealed that CD122Ab treatment significantly increased the proportion of PD-L1^+^ neutrophils, whereas adoptive transfer of CD8 Tregs suppressed PD-L1 expression (Supplemental Figure 9H). Moreover, PD-L1^+^ neutrophils exhibited lower CD62L expression compared with their PD-L1^–^ counterparts, further supporting the role for PD-L1 in modulating neutrophil lifespan (Figure 5M).

Previous studies have reported that upregulation of PD-L1 promotes the accumulation of neutrophils in the bone marrow (48). In addition, PD-L1 expression has been shown to protect cells from macrophage-mediated phagocytosis (49). In light of these findings, we hypothesized that the increase in peripheral PD-L1^+^ neutrophils following CD122Ab treatment might similarly result from reduced phagocytic clearance. We therefore examined the major sites of neutrophil clearance after MCAO. By using in vivo fluorescent antibody labeling, we were able to detect phagocytes engaged in neutrophil clearance, as indicated by the fluorescent signal (Figure 5N). The results showed that fluorescently labeled phagocytes were more abundant in the bone marrow than in the liver or spleen, indicating that the bone marrow is the primary site of neutrophil clearance after MCAO (Figure 5N). We further found that adoptive transfer of CD8 Tregs reduced the proportion of PD-L1^+^ neutrophils in the bone marrow (Supplemental Figure 9I). This finding suggests that PD-L1 expression may impair neutrophil clearance in the bone marrow.

We then intravenously administered a PD-L1 blocking antibody (PD-L1Ab) to mice in order to disrupt this pathway. PD-L1Ab treatment significantly enhanced the phagocytic activity of bone marrow phagocytes toward neutrophils (Figure 5O). In addition, PD-L1Ab treatment improved neurological recovery after MCAO, increased CD62L expression on peripheral neutrophils, and reduced both circulating neutrophil numbers and the proportion of hypersegmented neutrophils (Figure 5P and Supplemental Figure 9, J–N). Moreover, PD-L1 blockade alleviated HT and reduced BBB disruption after stroke (Supplemental Figure 9, O and P). Under PD-L1 blockade, however, adoptive transfer of CD8 Tregs failed to further increase CD62L expression or further reduce neutrophil abundance and hypersegmentation (Figure 5P and Supplemental Figure 9, J–N). Similarly, no additional protective effects on HT or BBB integrity were observed following CD8 Treg transfer in PD-L1Ab–treated mice (Supplemental Figure 9, O and P).

Collectively, these findings suggest that CD8 Tregs regulate neutrophil lifespan through modulation of glycolysis and PD-L1–mediated suppression of phagocytic clearance.

### HIF-1α is a central mediator of CD8 Treg–driven regulation of neutrophil metabolism and lifespan.

To dissect how CD8 Tregs regulate neutrophil glycolysis and PD-L1 expression, we intersected CD8 Treg–modulated genes with those linked to glycolysis and PD-L1 pathways, identifying 2 transcription factors: *Hif1a* and *Nfkb1* (Supplemental Figure 10A). However, in our long-term culture model, we observed that *Hif1a* expression changed significantly over time, showing a rapid increase at 24 hours, whereas *Nfkb1* expression remained largely unchanged (Figure 6A). These findings suggest that *Hif1a* may represent a more critical regulatory factor governing neutrophil lifespan. We therefore focused on *Hif1a* and examined changes at the protein level during long-term culture. The results showed that HIF-1α expression increased markedly at 24 hours relative to freshly isolated neutrophils, then showed a slight decline at 48 hours, but remained significantly elevated compared with baseline (Figure 6B). In addition, scRNA-seq analysis revealed that CD122Ab treatment induced elevated *Hif1a* expression in neutrophils (Figure 6C). To validate this in vivo, we examined HIF-1α protein levels in neutrophils from MCAO mice and found that CD122Ab significantly increased HIF-1α expression in both peripheral and brain-infiltrating neutrophils, whereas CD8 Treg transfer markedly reduced its expression (Figure 6D and Supplemental Figure 10B). These results suggest that CD8 Tregs may regulate the long-lived state of neutrophils, at least in part, through modulation of HIF-1α.

To further validate the functional role of HIF-1α, we crossed *Ly6g*-Cre mice with *Hif1a^fl/fl^* mice to generate neutrophil-specific *Hif1a*-knockout mice (*Ly6g*-Cre *-Hif1a^fl/fl^*, hereafter referred to as HIF-1α–KO mice) and further evaluated the effects of CD8 Treg adoptive transfer in this genetic background (Figure 6E). We first assessed neurological recovery after MCAO and found that HIF-1α–KO mice exhibited significantly improved neurological outcomes compared with WT mice (Supplemental Figure 10C). In parallel, HIF-1α deficiency markedly alleviated HT and reduced BBB disruption, as reflected by decreased endogenous IgG extravasation, reduced leakage of intravenously administered dextran, and increased coverage of ZO-1^+^ endothelial areas at day 3 after MCAO. Notably, adoptive transfer of CD8 Tregs did not confer additional protection against HT or BBB injury in HIF-1α–KO mice (Figure 6F and Supplemental Figure 10, D–F).

We next analyzed neutrophil counts and CD62L expression and found that HIF-1α deletion significantly reduced peripheral neutrophil numbers, accompanied by elevated CD62L expression and a reduced proportion of hypersegmented neutrophils, collectively indicating a shortened neutrophil lifespan (Figure 6, G–I). Furthermore, adoptive transfer of CD8 Tregs into HIF-1α–KO mice produced no additional changes in neutrophil numbers, CD62L expression, or the proportion of hypersegmented neutrophils (Figure 6, G–I), suggesting that CD8 Tregs regulate neutrophil abundance and lifespan primarily through suppression of HIF-1α.

In addition, we examined metabolic alterations and PD-L1 expression in neutrophils. Neutrophil-specific deletion of *Hif1a* markedly suppressed glycolytic activity (Figure 6J and Supplemental Figure 10G) and reduced the proportion of PD-L1^+^ neutrophils in both the peripheral blood and bone marrow (Figure 6, K and L). Consistent with the above findings, adoptive transfer of CD8 Tregs did not exert additional effects in the HIF-1α–KO background (Figure 6, J–L, and Supplemental Figure 10G), supporting HIF-1α as a key mediator of CD8 Treg–dependent regulation of neutrophil glycolysis and PD-L1 expression.

Finally, we investigated potential upstream mechanisms by which CD8 Tregs regulate HIF-1α expression in neutrophils. Given that HIF-1α is regulated by multiple upstream pathways (43, 50, 51), we analyzed differentially expressed genes between neutrophils cultured alone and those cocultured with CD8 Tregs and identified significantly higher *Tlr2* expression in neutrophils cultured alone (Figure 6M). Since the TLR2 pathway has been regarded as an important regulator of neutrophil function as well as HIF-1α expression (52–54), we next focused on its potential involvement in mediating CD8 Treg–dependent regulation. At day 3 after MCAO, TLR2 expression examination revealed that adoptive transfer of CD8 Tregs markedly decreased its levels in neutrophils from both peripheral blood and the ischemic brain (Figure 6N). To further validate the role of TLR2, we used *Tlr2*^–/–^ mice. TLR2 deficiency significantly reduced peripheral neutrophil numbers and decreased HIF-1α expression in circulating neutrophils (Figure 6O). Notably, adoptive transfer of CD8 Tregs further reduced peripheral neutrophil abundance and further suppressed HIF-1α expression in *Tlr2*^–/–^ mice (Figure 6O). These findings suggest that, although TLR2 plays an important role in maintaining stable HIF-1α expression, CD8 Tregs may also regulate HIF-1α through additional pathways. We also explored potential cytokines or mediators produced by CD8 Tregs that might contribute to the regulation of neutrophils. scRNA-seq analysis revealed that CD8 Tregs, compared with other T cell subsets, exhibited characteristically higher expression of *Anxa2* (Supplemental Figure 10H). Consistently, flow cytometric analysis of peripheral blood from MCAO mice confirmed that CD8 Tregs expressed higher levels of ANXA2 than other T cells (Supplemental Figure 10I). Given that ANXA2 has been reported to functionally interact with TLR2 in neutrophils (55), these findings suggest that the ANXA2 may participate in the regulatory effects of CD8 Tregs on neutrophil HIF-1α expression.

Collectively, these findings suggest that CD8 Tregs regulate neutrophil lifespan through suppression of HIF-1α expression.

### The CD8 Treg–neutrophil regulatory axis is preserved in aged mice after stroke.

Elderly individuals represent a major population affected by ischemic stroke, and aging-associated immune dysregulation has been reported to contribute to worse neurological outcomes after stroke (36, 56, 57). In our previous studies, we found that aging promotes peripheral neutrophil senescence, accompanied by increased brain infiltration and aggravated BBB disruption after stroke (58). Based on these findings, we further analyzed peripheral neutrophil subsets in aged mice and found that the proportions of the long-lived neutrophil subsets Neu3 and Neu4 were markedly increased in the peripheral blood of aged MCAO mice (Supplemental Figure 11, A–C).

More importantly, we previously observed a significant reduction in CD8 Treg abundance in aged mice, suggesting that the regulatory balance between CD8 Tregs and neutrophils may be further disrupted under aging conditions after MCAO (58). To determine whether restoring CD8 Treg abundance could rescue this imbalance, we adoptively transferred CD8 Tregs into aged MCAO mice and found that CD8 Treg transfer significantly reduced neutrophil numbers (Supplemental Figure 11, D and E). Following CD8 Treg transfer, neutrophils from aged MCAO mice exhibited significantly increased CD62L expression, accompanied by marked reductions in PD-L1 and HIF-1α expression (Supplemental Figure 11, F–H). Importantly, adoptive transfer of CD8 Tregs also alleviated HT and attenuated BBB disruption in aged mice after stroke (Supplemental Figure 11, I and J).

Collectively, these findings demonstrate that the CD8 Treg–neutrophil regulatory axis is conserved in aged mice and that restoring CD8 Treg abundance may represent a potential therapeutic strategy for mitigating HT in elderly stroke patients.

### Human CD8 Tregs regulate neutrophil lifespan via mechanisms analogous to those observed in mice.

Finally, we sought to determine whether a similar regulatory process occurs in human cells. Peripheral blood samples were collected from patients with ischemic stroke, and neutrophils were isolated to assess alterations during long-term culture. Davis and colleagues have reported that KIR molecules serve as key markers of human CD8 Tregs, which are functionally equivalent to Ly49 receptors in mice (59). Based on this, we isolated human CD8 Tregs by flow cytometric sorting and cocultured them with neutrophils to examine their effects on the long-lived state of human neutrophils (Supplemental Figure 12, A and B). Results from the long-term culture model revealed that CD62L^lo^ neutrophils, representing the long-lived state, increased rapidly within the first 24 hours and then rose more gradually between 24 and 48 hours, accompanied by progressive upregulation of HIF-1α and PD-L1 expression (Supplemental Figure 12, C–F). In addition, CD62L^lo^ neutrophils expressed higher levels of HIF-1α and PD-L1 than CD62L^hi^ cells at 48 hours (Supplemental Figure 12, G and H). Moreover, both HIF-1α and PD-L1 were significantly upregulated in CD62L^lo^ neutrophils at 48 hours relative to 0 hours (Supplemental Figure 12, G and H). These findings indicate that the lifespan extension process of patient-derived neutrophils shares key features with that observed in mice.

We next cocultured human CD8 Tregs with neutrophils and found that CD8 Treg coculture significantly reduced the proportion of CD62L^lo^ neutrophils, accompanied by decreased expression of HIF-1α and PD-L1 in neutrophils (Supplemental Figure 12, I–K). In addition, the glycolytic capacity of neutrophils was suppressed when cocultured with CD8 Tregs (Supplemental Figure 12L). We also examined ANXA2 expression in human CD8 Tregs and found significantly higher expression than in other T cell subsets (Supplemental Figure 12M). These findings suggest that human CD8 Tregs may employ mechanisms analogous to those of murine CD8 Tregs in modulating neutrophil lifespan.

At last, to examine the impact of CD8 Treg–mediated regulation of neutrophil lifespan on endothelial barrier integrity, we isolated brain microvascular endothelial cells from patients undergoing temporal lobectomy and established an in vitro OGD model (Supplemental Figure 12N). Neutrophils, either cultured alone or preconditioned by coculture with CD8 Tregs, were added to the OGD-treated endothelial cells (Supplemental Figure 12O). The results showed that neutrophils cocultured with CD8 Tregs exhibited reduced transendothelial migration and decreased dextran leakage (Supplemental Figure 12, P–R). These findings suggest that human CD8 Tregs may regulate neutrophil lifespan and thereby exert a potential protective effect on endothelial barrier integrity.

## Discussion

Neutrophils were traditionally regarded as a relatively homogeneous population, but advances in scRNA-seq and other high-dimensional technologies have revealed substantial neutrophil heterogeneity across tissues (60–62). In our study, we focused on the changes occurring within circulating neutrophils. By depleting or transferring CD8 Tregs, we found that the circulating milieu itself is capable of influencing neutrophil states. Although such interventions did not generate completely distinct subpopulations, they induced a shift in the overall neutrophil pool. Specifically, CD8 Treg depletion increased the proportion of long-lived terminal neutrophils without affecting bone marrow proliferation, neutrophil release, or detectable apoptosis in the circulation. Current concepts emphasize that circulating neutrophil homeostasis is maintained through a highly dynamic turnover process involving bone marrow release, tissue recruitment, reverse migration, homing, cell death, and phagocytic clearance (63, 64). Within this framework, alterations in circulating neutrophil abundance may not necessarily reflect changes in apoptosis at a single time point, but instead represent the integrated outcome of multiple dynamic processes governing neutrophil turnover. Our findings therefore highlight the importance of systemic immune regulation in shaping circulating neutrophil turnover and underscore the need to directly investigate neutrophil dynamics in vivo.

CD8 Tregs exhibited marked transcriptional changes at day 1 after MCAO, including upregulation of apoptosis-related pathways. As no substantial differences in immune functional programs were observed between HT and NHT CD8 Tregs, their more pronounced reduction in HT mice may be associated with enhanced apoptosis. This differential apoptosis may reflect interindividual variation in the CD8 Treg response to stroke. Importantly, HT mice already exhibited elevated CD8 Treg apoptosis at day 1 after MCAO compared with NHT mice, and given that HT progressively develops over the first 3 days after MCAO (65, 66), this early apoptotic increase may represent a potential predisposing factor for HT development. It is widely accepted that poststroke neuroendocrine responses induce profound systemic immune alterations, leading to a state of stroke-induced immunosuppression (67). Differential neuroendocrine responses following stroke may therefore represent an important contributor to the interindividual heterogeneity in CD8 Treg dynamics observed in this study. Given that human CD8 Tregs are likely to exhibit even greater heterogeneity owing to diverse clinical and environmental factors, future studies are needed to determine whether differences in stress hormone responses contribute to this heterogeneity in adaptive immune regulation. Such findings may help facilitate early immunomodulatory interventions and influence therapeutic decision-making after stroke.

Our findings primarily support a peripheral site of CD8 Treg–mediated neutrophil regulation during the acute phase of stroke, largely attributable to the limited T cell infiltration into the brain during this period, which constrains the potential for local CNS regulation by CD8 Tregs. Nevertheless, it is worth noting that neutrophils, macrophages, and microglia represent the 3 predominant immune cell populations within the brain during the acute phase of stroke. Given the potent phagocytic activity of macrophages and microglia, whether these cells modulate neutrophil lifespan within the CNS and thereby influence HT and stroke pathology warrants further investigation. Furthermore, during the subacute and chronic phases of stroke, T cell infiltration into the brain progressively increases (68). Whether the subsequent accumulation of CD8 Tregs within the CNS may exert regulatory effects on the intracerebral immune microenvironment during these later stages represents an important question for future research.

In this study, we used KIRs as defining markers of human CD8 Tregs. KIR^+^ CD8 T cells have been reported to suppress T cell function, supporting their broad immunoregulatory roles across autoimmune, infectious, inflammatory, and neoplastic diseases (59, 69). By linking KIR^+^ CD8 T cells to the regulation of neutrophil lifespan, our study extends their functional scope and highlights a previously underappreciated dimension of their immunomodulatory capacity.

This study has several limitations. First, we did not further investigate how peripheral alterations in neutrophil lifespan influence the intracerebral immune microenvironment during the acute phase of stroke. Second, although CD8 Tregs regulate neutrophil lifespan and HT, anti-CD122 treatment may also affect other CD122-expressing immune cells and contribute to HT through neutrophil-independent mechanisms. Furthermore, it remains unclear whether PD-L1 expression on neutrophils might, in turn, modulate T cell function.

In summary, our study identifies CD8 Tregs as key regulators of circulating neutrophil homeostasis after ischemic stroke. CD8 Tregs shortened neutrophil lifespan through HIF-1α–dependent metabolic regulation and enhanced neutrophil clearance, thereby protecting the BBB and reducing HT. These findings provide new insights into peripheral immune regulation and potential therapeutic strategies for ischemic stroke.

## Methods

Additional details may be found in Supplemental Methods.

### Sex as a biological variable.

The human cohort included both male and female patients. Only male mice were used in the animal experiments to minimize variability related to sex-specific differences. The potential influence of sex on the observed immune mechanisms in animal models warrants further investigation.

### Patients.

A total of 222 patients with ischemic stroke and 4 patients undergoing temporal lobectomy were included for clinical sample collection and endothelial cell isolation, respectively. Detailed eligibility criteria, clinical characteristics, and procedures are provided in the Supplemental Methods and Supplemental Table 1.

### Mice and MCAO model.

Detailed information on mouse strains, experimental procedures, and the MCAO model is provided in the Supplemental Methods.

### Statistics.

Data are presented as mean ± SD or SEM. Statistical analyses were performed using 2-tailed Student’s *t* tests, 1- or 2-way ANOVA, mixed effects models, or Wilcoxon’s rank-sum tests, as appropriate, using R (v4.4.3) or GraphPad Prism (v10.4.0). *P* < 0.05 was considered significant.

### Study approval.

We collected clinical samples from the Second Affiliated Hospital, Zhejiang University School of Medicine, with written informed consent and approval from the Institutional Review Board. All animal experiments in this study were approved by the Institutional Ethics Committee of the Second Affiliated Hospital, Zhejiang University School of Medicine.

### Data availability.

The bulk RNA-seq and single-cell RNA-seq data generated in this study have been deposited in the NCBI Gene Expression Omnibus (GEO) under accession numbers GSE343620, GSE343642, respectively. Human RNA-seq data have been deposited in the Genome Sequence Archive for Human (GSA-Human) under accession number HRA013553 and are available under controlled access to protect participant privacy and confidentiality. Data access requests can be submitted to the Human neutrophil project Data Access Committee (DAC; HDAC006853; contact: Ligen Shi). Supporting data values for all quantitative results are provided in the Supporting Data Values XLS file. Additional data and analytic code supporting this study are available from the corresponding author upon reasonable request.

## Author contributions

Conceptualization, LS, JZ, and JY; investigation, JL, QZ, JC, HL, and AP; software, JL and ZJ; formal analysis, JL, XZ, YG, XD, and GZ; writing – original draft, JL, ZC, YQ, and JY; writing – review and editing, JZ and LS; supervision, JZ and LS; visualization, JL, QZ, JC, and HL; funding acquisition, JL, JZ, and LS. The order of the co–first authors (JL, QZ, and JC) was determined by the relative extent of their experimental and analytical contributions to this study.

## Conflict of interest

The authors have declared that no conflict of interest exists.

## Funding support

- The National Natural Science Foundation of China (82322022 and 82471296 to LS; 82471295 and 82271301 to JZ).
- The Natural Science Foundation of Zhejiang Province (LR23H250001 and LQ21H090012 to LS).
- The “Pioneer” and “Leading Goose” R&D Program of Zhejiang (2026C02A1178 to LS).
- The Postdoctoral Fellowship Program of CPSF (GZC20251419 to JL).

## Supplementary Material

Supplemental data

Supplemental table 1

Supplemental table 2

Supplemental table 3

Supplemental table 4

Supporting data values

## Figures and Tables

**Figure 1:**
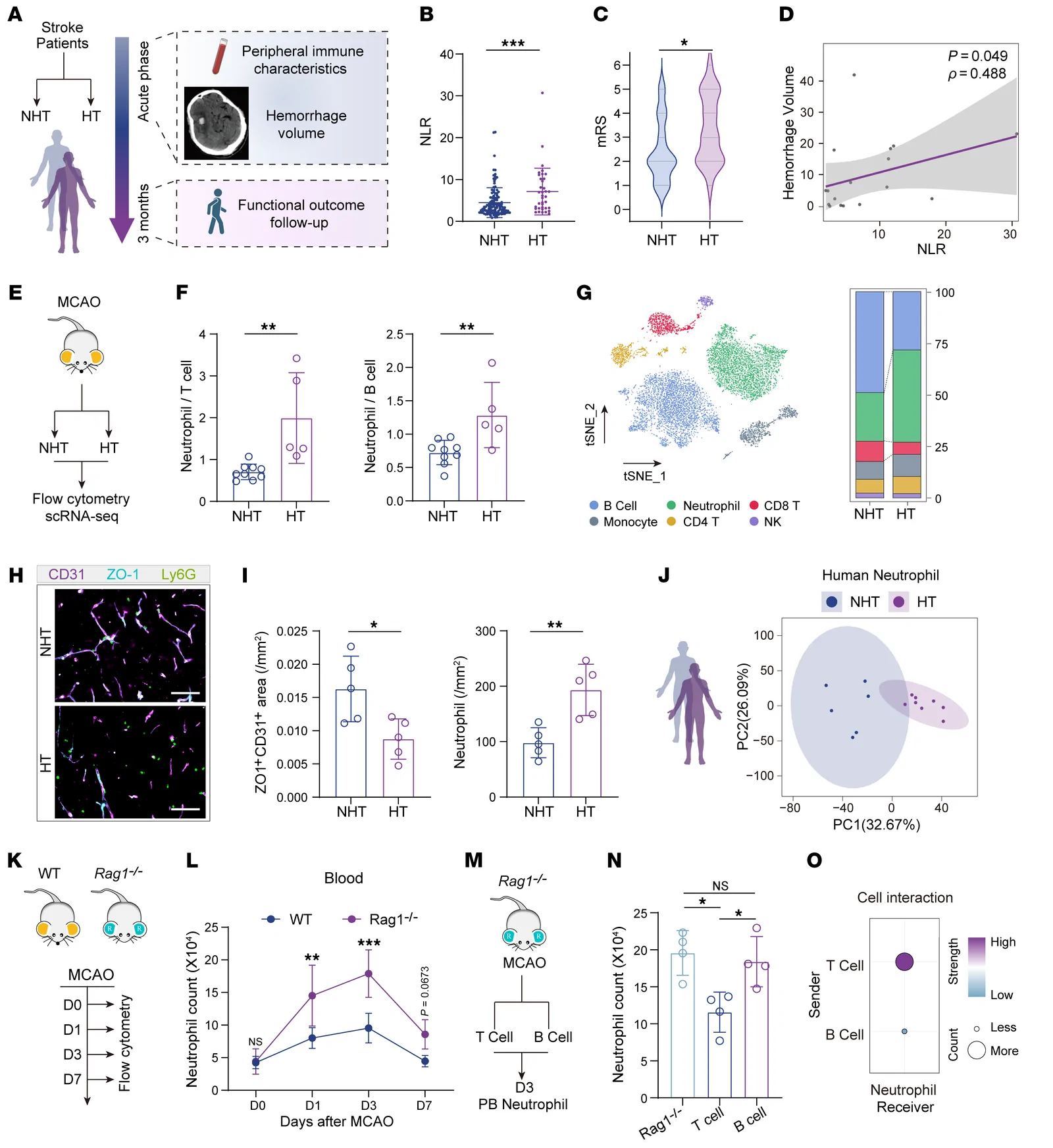
Elevated NLR in patients with HT reflects impaired T cell–mediated regulation of neutrophils. (**A**) Workflow for patient sample analysis. (**B**) NLR in patients with HT (*n* = 37) and NHT (*n* = 120). (**C**) Three-month mRS scores in patients with HT and NHT (*n* = 21/group). (**D**) Correlation between NLR and hematoma volume in patients with parenchymal hematoma (*n* = 17), adjusted for age and hypertension history. (**E**) Workflow for peripheral immune profiling of HT and NHT mice at day 3 after MCAO. (**F**) Peripheral blood neutrophil/T cell (left) and neutrophil/B cell (right) ratios in HT (*n* = 5) and NHT (*n* = 9) mice. (**G**) t-SNE of peripheral immune cells (left) and cluster proportions (right) in HT and NHT mice at day 3 after MCAO. (**H** and **I**) CD31/Ly6G/ZO-1 staining (**H**; scale bar: 100 μm) and ZO-1^+^ endothelial coverage and neutrophil infiltration (**I**; *n* = 5/group) in HT and NHT brains. (**J**) PCA of peripheral neutrophil transcriptomes from patients with HT and NHT. (**K**) Workflow for peripheral immune cell analysis in WT and *Rag1^–/–^* mice after MCAO. (**L**) Peripheral blood neutrophils in WT and *Rag1^–/–^* mice (*n* = 5/group). (**M**) Workflow for T or B cell transfer into *Rag1^–/–^* mice after MCAO. (**N**) Peripheral neutrophil counts following T or B cell transfer (*n* = 4/group). (**O**) Regulatory interactions of T and B cells with neutrophils as the receiver population. Data are presented as mean ± SD. *P < 0.05, **P < 0.01, ***P < 0.001. Mann-Whitney *U* test (**B** and **C**), Student’s *t* test (**F** and **I**), 1-way ANOVA (**N**) or 2-way ANOVA (**L**) with Tukey’s post hoc test.

**Figure 2:**
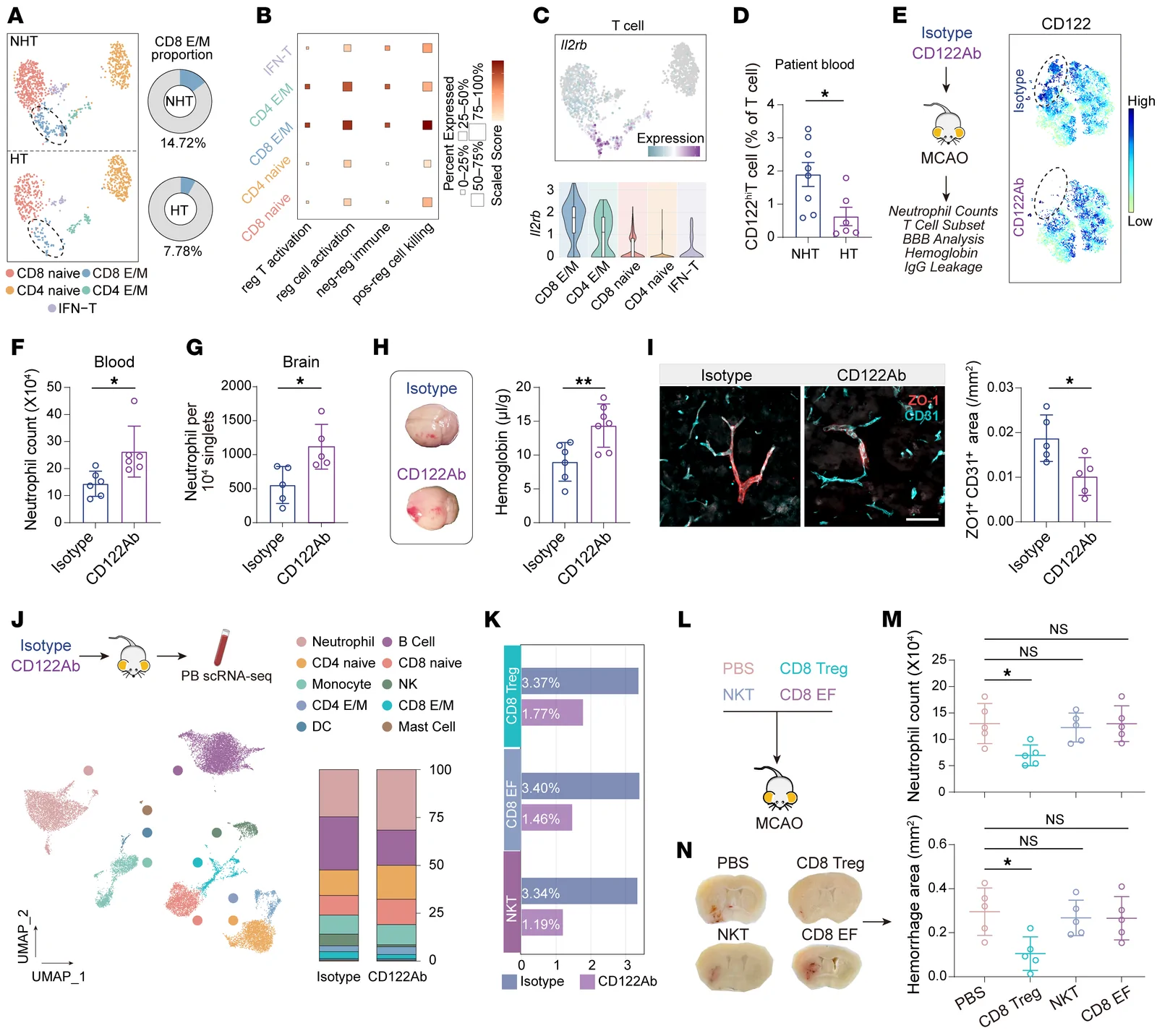
CD8 Tregs regulate neutrophil numbers. (**A**) UMAP of T cells from HT and NHT mice (left) and CD8 E/M proportions (right). (**B**) Immunoregulatory functions of T cell subsets. reg cell activation, regulation of cell activation; reg T activation, regulation of α-β T cell activation; neg-reg immune, negative regulation of immune system process; pos reg cell killing, positive regulation of cell killing. (**C**) Feature (top) and violin (bottom) plots of *Il2rb* expression. (**D**) Peripheral CD122^hi^ T cells in patients with HT (*n* = 6) and NHT (*n* = 8). (**E**) Workflow for isotype or CD122Ab treatment (left) and T cell CD122 expression (right). (**F** and **G**) Peripheral (**F**; *n* = 6/group) and brain-infiltrating (**G**; *n* = 5/group) neutrophils following CD122Ab treatment. (**H**) Cerebral hemoglobin levels in isotype (*n* = 6) and CD122Ab-treated (*n* = 7) mice. (**I**) CD31/ZO-1 staining and ZO-1^+^ endothelial coverage (*n* = 5/group; scale bar: 100 μm). (**J**) scRNA-seq workflow, UMAP (left), and immune cell proportions (right) following isotype or CD122Ab treatment. (**K**) Proportions of CD8 Tregs, CD8 EF, and NKT cells among peripheral T cells. (**L**) Workflow for CD8 Treg, CD8 EF, or NKT cell transfer after MCAO. (**M** and **N**) Peripheral neutrophil counts (**M**) and hemorrhage (**N**) following PBS, CD8 Treg, CD8 EF, or NKT transfer (*n* = 5/group). Data are presented as mean ± SD (**F**–**I**, **M**, and **N**) or mean ± SEM (**D**). *P < 0.05, **P < 0.01. Student’s *t* test (**D**, **F**–**I**), 1-way ANOVA followed by Tukey’s multiple comparisons test (**M** and **N**).

**Figure 3:**
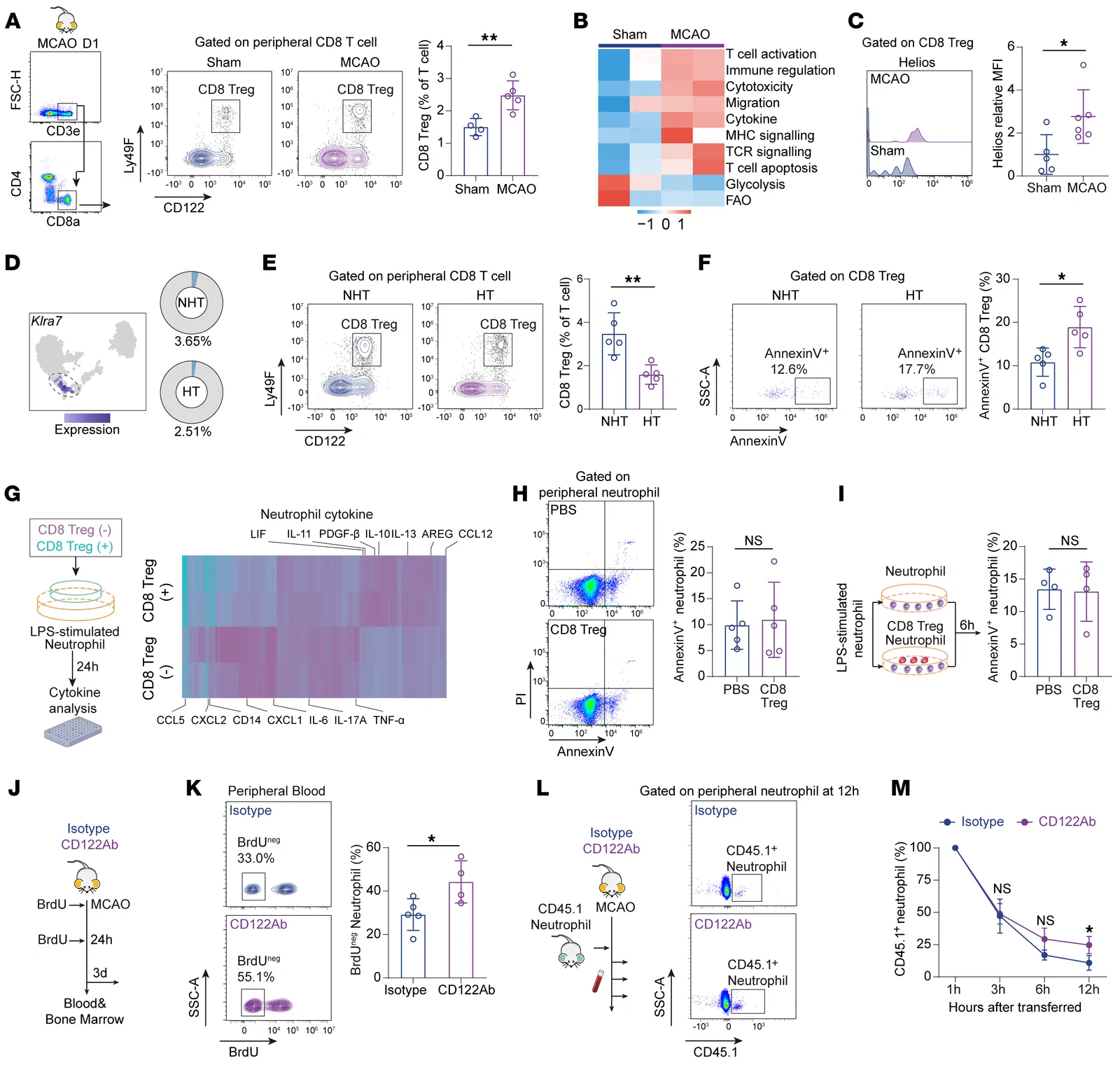
Reduction in CD8 Treg abundance and its regulation of neutrophil lifespan following stroke. (**A**) Peripheral CD8 Tregs at day 1 after MCAO (sham, *n* = 4; MCAO, *n* = 5). (**B**) Functional gene signatures in peripheral CD8 Tregs from sham and MCAO mice at day 1 by bulk RNA-seq. (**C**) Helios expression in peripheral CD8 Tregs at day 1, normalized to the sham group (sham, *n* = 5; MCAO, *n* = 6). (**D**) *Klra7* expression across peripheral T cell clusters (left) and CD8 Treg proportions in HT and NHT mice (right). (**E** and **F**) Peripheral CD8 Treg proportions (**E**) and Annexin V^+^ CD8 Tregs (**F**) in NHT and HT mice at day 3 after MCAO (*n* = 5/group). (**G**) CD8 Treg-neutrophil coculture model and neutrophil cytokine expression (*n* = 2/group). (**H**) Annexin V^+^ peripheral blood neutrophils in the PBS and CD8 Treg groups (*n* = 5/group). (**I**) Neutrophil apoptosis after culture alone or with CD8 Tregs under LPS stimulation (*n* = 4/group). (**J** and **K**) BrdU-labeling workflow (**J**) and peripheral BrdU^neg^ neutrophils (**K**) in isotype (*n* = 5) and CD122Ab-treated (*n* = 4) mice. (**L** and **M**) CD45.1^+^ neutrophil transfer (**L**) and circulating CD45.1^+^ neutrophils over time (**M**; *n* = 4/group/time point), with values normalized to each mouse’s baseline at 1 hour. Data are presented as mean ± SD. *P < 0.05, **P < 0.01. Student’s *t* test (**A**, **C**, **E**, **F**, **H**, **I**, and **K**), 2-way ANOVA (**M**) with Tukey’s test.

**Figure 4:**
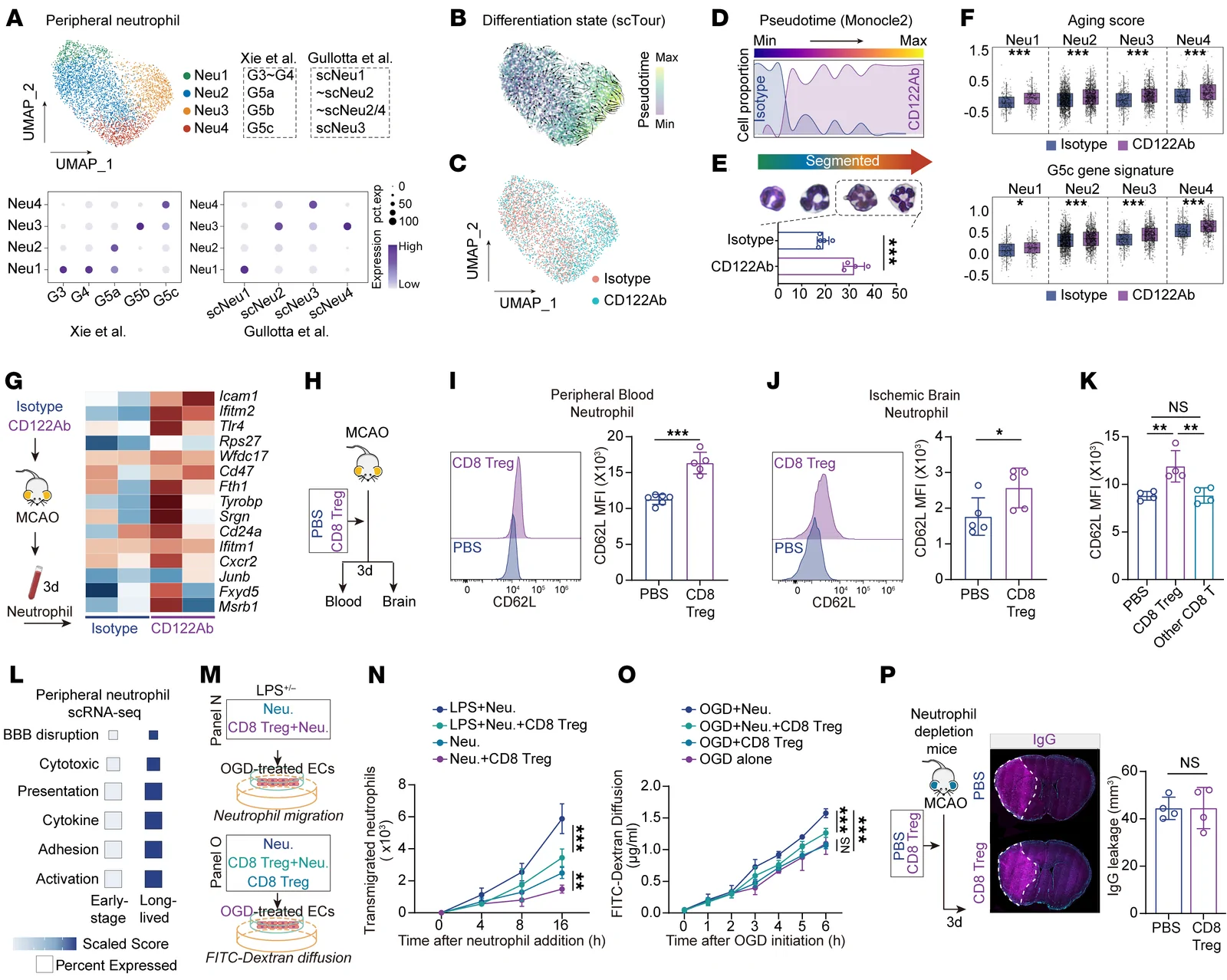
CD8 Tregs regulate neutrophil lifespan. (**A**) UMAP of neutrophils from isotype and CD122Ab-treated mice (top) and similarity to reported peripheral neutrophil subclusters (bottom). (**B**) Neutrophil differentiation states inferred by scTour. (**C**) Distribution of isotype and CD122Ab-treated neutrophils on UMAP. (**D**) Neutrophil distribution along Monocle2 pseudotime. (**E**) Nuclear morphology and hypersegmented neutrophils (isotype, *n* = 5; CD122Ab, *n* = 4). (**F**) Aging scores (top) and G5c signatures (bottom) across neutrophil subclusters. (**G**) Late neutrotime gene expression in peripheral neutrophils from isotype and CD122Ab-treated mice at day 3 after MCAO (*n* = 2/group). (**H**) Workflow for CD8 Treg transfer and neutrophil analysis at day 3 after MCAO. (**I** and **J**) CD62L expression on peripheral (I; PBS, *n* = 6; CD8 Treg, *n* = 5) and brain-infiltrating (J; *n* = 5/group) neutrophils. (**K**) CD62L expression on neutrophils cultured alone or with CD8 Tregs or other CD8 T cells (*n* = 4/group). (**L**) Functional characteristics of early-stage (Neu1/Neu2) and long-lived (Neu3/Neu4) neutrophils. (**M**) Schematic illustration of the OGD endothelial barrier model. (**N** and **O**) Neutrophil transendothelial migration (**N**) and FITC-dextran leakage (**O**) over time (*n* = 3/group). For **N**, 0 hours represents neutrophil addition. (**P**) IgG leakage following CD8 Treg transfer in neutrophil-depleted MCAO mice (*n* = 4/group). Data are mean ± SD. *P < 0.05, **P < 0.01, ***P < 0.001. Student’s *t* test (**E**, **I**, **J**, and **P**), 1-way ANOVA (**K**), 2-way ANOVA (**N**, **O**) with Tukey’s multiple comparisons test, or Bonferroni-corrected Wilcoxon rank-sum test (**F**).

**Figure 5:**
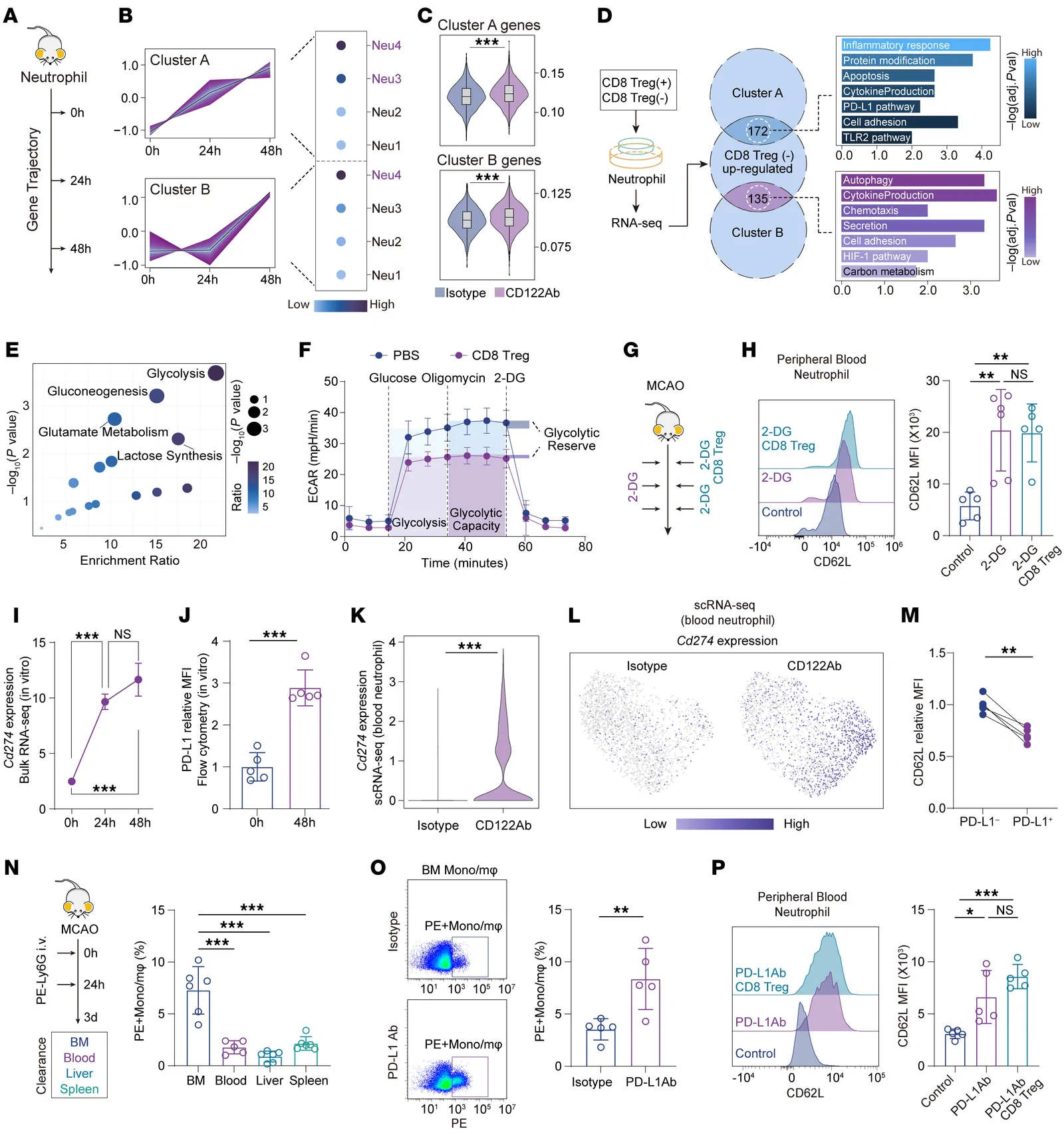
CD8 Tregs regulate neutrophil lifespan through modulation of glycolysis and PD-L1 expression. (**A**) RNA-seq workflow for long-term neutrophil culture. (**B**) Temporal expression of Cluster A and B genes (left; Supplemental Table 3) and their distribution across neutrophil subclusters (right). (**C**) Cluster A and B gene expression in neutrophils from isotype and CD122Ab-treated mice. (**D**) Intersection of coculture differentially expressed genes with Cluster A/B signatures (left) and pathway enrichment of core genes (right). (**E**) Metabolic pathways enriched in neutrophils cultured alone versus cocultured with CD8 Tregs. (**F**) ECAR curves in neutrophils cultured alone or with CD8 Tregs (*n* = 6/group). (**G**) Experimental design for 2-DG treatment. (**H**) Peripheral neutrophil CD62L expression following 2-DG treatment with or without CD8 Treg transfer (control and 2-DG + CD8 Treg, *n* = 5/group; 2-DG, *n* = 6). (**I**) *Cd274* expression during long-term neutrophil culture (bulk RNA-seq; *n* = 3/time point). (**J**) PD-L1 expression in freshly isolated and 48-hour–cultured neutrophils (*n* = 5/group). (**K** and **L**) *Cd274* expression in neutrophils from isotype and CD122Ab-treated mice shown by violin (**K**) and feature plots (**L**). (**M**) Relative CD62L MFI in peripheral PD-L1^+^ and PD-L1^–^ neutrophils, normalized to mean PD-L1^–^ MFI (*n* = 5/group). (**N**) Intravenous PE-Ly6G labeling (left) and PE^+^ monocytes/macrophages in bone marrow (*n* = 6), blood (*n* = 5), liver (*n* = 6), and spleen (*n* = 6) (right). (**O**) Bone marrow PE^+^ monocytes/macrophages following isotype or PD-L1Ab treatment (*n* = 5/group). (**P**) Peripheral neutrophil CD62L expression across groups (*n* = 5/group). Data are presented as mean ± SD. *P < 0.05, **P < 0.01, ***P < 0.001. Student’s *t* test (**J** and **O**), paired Student’s *t* test (**M**), 1-way ANOVA followed by Tukey’s multiple comparisons test (**H**, **N**, and **P**), DESeq2 differential expression analysis (**I**), Bonferroni-corrected Wilcoxon rank-sum test (**C** and **K**).

**Figure 6:**
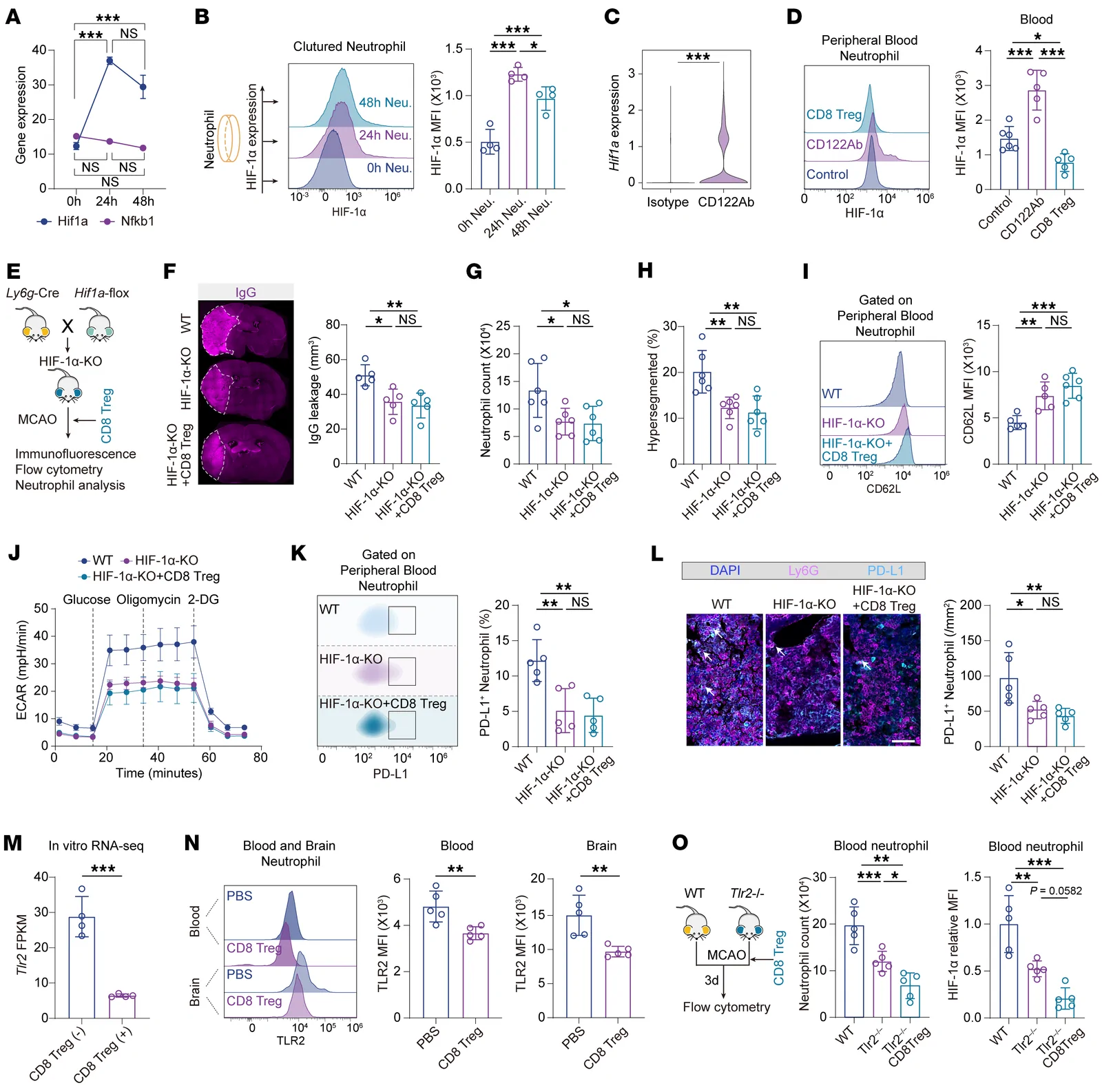
HIF-1α mediates the regulatory effects of CD8 Tregs on neutrophils. (**A**) *Hif1a* and *Nfkb1* expression during long-term neutrophil culture (bulk RNA-seq; *n* = 3/time point). (**B**) Neutrophil HIF-1α expression over time (*n* = 4/time point). (**C**) *Hif1a* expression in neutrophils from isotype and CD122Ab-treated mice. (**D**) HIF-1α expression in peripheral neutrophils (control, *n* = 6; CD122Ab and CD8 Treg, *n* = 5). (**E**) Generation of *Ly6g*-Cre *-Hif1a^fl/fl^* mice and experimental design. (**F**) Brain IgG leakage in WT, HIF-1α–KO, and HIF-1α–KO + CD8 Treg mice (*n* = 5/group). (**G** and **H**) Peripheral neutrophil counts (**G**; *n* = 6/group) and hypersegmented neutrophils (**H**; *n* = 6/group) across groups. (**I**) Peripheral neutrophil CD62L expression (WT and HIF1α-KO, *n* = 5; HIF1α-KO + CD8 Treg, *n* = 6). (**J**) ECAR curves of WT neutrophils (*n* = 4), HIF1α-KO neutrophils (*n* = 5), and HIF-1α KO neutrophils cocultured with CD8 Tregs (*n* = 5). (**K**) PD-L1^+^ neutrophils across groups (*n* = 5/group). (**L**) Bone marrow PD-L1^+^ neutrophils (*n* = 5/group; scale bar: 50 μm). (**M**) *Tlr2* expression in neutrophils from the in vitro coculture RNA-seq dataset (bulk RNA-seq; *n* = 4). (**N**) TLR2 expression in peripheral and brain-infiltrating neutrophils (*n* = 5/group). (**O**) Experimental design for WT, *Tlr2*^–/–^, and *Tlr2*^–/–^ mice receiving CD8 Treg transfer (left), peripheral neutrophil counts (middle), and HIF-1α expression normalized to the WT group (right; *n* = 5/group). Data are presented as mean ± SD. *P < 0.05, **P < 0.01, ***P < 0.001. Student’s *t* test (**N**), 1-way ANOVA followed by Tukey’s multiple comparisons test (**B**, **D**, **F**–**I**, **K**, **L**, and **O**), DESeq2 differential expression analysis (**A** and **M**), Bonferroni-corrected Wilcoxon rank-sum test (**C**).

## Version 1 09/01/2026

In-Press Preview

## Version 2 10/15/2026

Electronic publication
