# Supplementary material for: CD8+ regulatory T cells shorten neutrophil lifespan to reduce hemorrhagic transformation after ischemic stroke
Source: J Clin Invest. 2026 Sep 1;136(20):e203868. doi: 10.1172/JCI203868 (PMC13668627; doi:10.1172/JCI203868)
Supplement: Supplementary file 1 [file jci-136-203868-s178.pdf]

List of Supplementary materials

Materials and Methods

Supplemental Figures 1-12

Supplemental References

Materials and Methods

Sex as a biological variable

The human cohort included both male and female patients. Only male mice were used in the animal experiments to minimize variability related to sex-specific differences. The potential influence of sex on the observed immune mechanisms in animal models warrants further investigation.

Patients

We collected clinical samples from the Second Affiliated Hospital, Zhejiang University School of Medicine, with written informed consent and approval from the Institutional Review Board. A total of 222 ischemic stroke patients were included in the study. The median age of the cohort was 67.2 years, consisting of 135 males and 87 females, with detailed demographic and clinical characteristics provided in **Table S1**. *Patient inclusion criteria were as follows: individuals who presented with clinical signs of ischemic stroke with either confirmation on diffusion-weighted MRI or absence of an alternative diagnosis on CT. HT was defined as the presence of intracranial hemorrhage within the infarcted area as confirmed by CT imaging. Patients with autoimmune diseases, acute infections, or hematological disorders that could affect neutrophil function were excluded from the study. 4 patients with bilateral hippocampal sclerosis who underwent temporal lobectomy were included in this study (***Table S1)***, and the resected temporal lobe tissue was used for subsequent isolation of brain microvascular endothelial cells.*

Mice

6-8-week-old male C57BL/6J mice were obtained from Beijing Vital River Laboratory Animal Technology Co. Ltd. (Beijing, China). Rag1$^{-/-}$, CD45.1, Tlr2$^{-/-}$, Foxp3 reporter mice, Ly6g-Cre and Hif1a$^{fl/fl}$ mice were purchased from Shanghai Model Organisms

Center. Neutrophil-specific Hif1a conditional knockout mice were generated by crossing Ly6g-Cre mice with Hif1a$^{fl/fl}$ mice. A total of 641 mice were used in this study. All mice were housed in a specific-pathogen-free facility under controlled environmental conditions (22 ± 2 °C; 40%–70% humidity) with a 12-hour light/dark cycle and ad libitum access to food and water. Animals were randomly allocated to experimental groups using a randomization protocol, and treatments were assigned by blinded investigators. Euthanasia was performed using carbon dioxide in accordance with institutional guidelines. All procedures complied with the National Institutes of Health Guide for the Care and Use of Laboratory Animals. All experimental protocols involving animals were reviewed and approved by the Institutional Ethics Committee of the Second Affiliated Hospital, Zhejiang University School of Medicine.

Clinical Data Collection

NLR was determined at admission using the first available complete blood count, calculated as the absolute neutrophil count divided by the absolute lymphocyte count. Functional outcomes were assessed at 3 months after stroke onset using the mRS. This ordinal scale ranges from 0 (no disability) and 1 (no significant disability despite mild symptoms) to 5 (severe disability and bedridden state) and 6 (death). The mRS score was evaluated by a certified rater blinded to treatment assignment. For patients unable to attend an in-person visit, structured telephone interviews were conducted to obtain the mRS score. Hemorrhage volume was calculated in patients who exhibited parenchymal hematoma within the infarcted region on CT, as defined by the ECASS and Heidelberg classification criteria. The volume was estimated using the following formula: (length × width × number of slices × slice thickness)/2.

Cell Isolation from Human

Neutrophil Isolation from Patient Blood

Neutrophil isolation from patient blood was performed as previously described (1). Briefly, peripheral blood samples from patients were collected into anticoagulant-coated tubes and centrifuged at 400 × g for 10 min. The red blood cell (RBC)-

containing layer was resuspended in 3% dextran, gently inverted, and allowed to sediment for 25 min. The supernatant was carefully aspirated, and residual RBCs were lysed using 33 mM NaCl. Lysis was terminated by restoring isotonicity with 267 mM NaCl, followed by centrifugation in Hank's Balanced Salt Solution (HBSS) to further reduce RBC contamination. Subsequently, the cell suspension was separated using a discontinuous Percoll density gradient (51% and 42%) centrifugation at 400 × g for 10 min, after which neutrophils were recovered as a pellet at the bottom of the tube. The isolated cells were washed, resuspended in HBSS, and counted prior to downstream experiments. The purified neutrophils were either used directly for RNA-seq or maintained in culture for subsequent functional assays.

CD8 Treg Isolation from Patient Blood

Peripheral blood was collected from patients, and PBMCs were isolated by Ficoll density gradient centrifugation. Cells were washed, incubated with antibodies against APC/Cyanine7 anti-human CD3 (UCHT1, BioLegend, 300425), PerCP/Cyanine5.5 anti-human CD8 (SK1, BioLegend, 344709), Brilliant Violet 421 anti-human CD122(IL-2Rβ) (TU27, BioLegend, 339009) and APC anti-huamn CD158b (DX27, BioLegend, 312611) and subsequently sorted on a flow cytometer to obtain human CD8 Treg cells (BD FACS Aria III).

Human Brain endothelial cell sorting

Human brain microvascular endothelial cells were obtained from resected temporal lobe tissue of epilepsy patients. Briefly, excised tissue was enzymatically dissociated and myelin was removed using the ABDK dissociation kit. The resulting single-cell suspension was incubated with antibodies against FITC Anti-Human CD45 (2D1, BD Biosciences, 572687) and BUV395 Anti-Human CD31 (WM59, BD Biosciences, 565290), followed by flow cytometric sorting to isolate CD45□CD31□ brain endothelial cells.

MCAO Models

MCAO models were established by endovascular occlusion of the left middle cerebral

artery for 60 min. Mice were anesthetized with 1% pentobarbital and maintained on a 30% O_2 / 70% N_2O mixture to allow spontaneous breathing. A monofilament with a silicon-coated tip (Doccol Corporation, MA, USA) was inserted into the external carotid artery (ECA) and advanced through the internal carotid artery (ICA) until resistance was felt, indicating occlusion of the MCA origin. The filament was left in place for 60 min to block cerebral blood flow and subsequently withdrawn to allow reperfusion. The residual end of the ECA was then ligated. Core body temperature was maintained at 37.0 ± 0.5 °C throughout the procedure using a heating pad, and mice were kept on a warming blanket until full recovery from anesthesia. Sham-operated mice underwent identical surgical exposure of the carotid triangle without MCA occlusion. Animals showing less than 70% reduction in regional cerebral blood flow (rCBF, measured by laser Doppler flowmetry) compared to baseline were excluded from the study. HT was assessed at the experimental endpoint during tissue harvest. Brains were macroscopically examined for visible hemorrhagic foci or hematoma formation on the brain surface and within ischemic regions. Mice with detectable hemorrhage were classified as the HT group, whereas mice without visible hemorrhage were classified as the NHT group. In this study, the overall mortality rate was 14.41% in MCAO-operated mice, 11.68% in NHT mice, and 18.30% in HT mice, while no mortality was observed in sham-operated mice.

Hemoglobin Content Measurement

Hemoglobin content was measured as previously described (2). Briefly, brains from sham-operated mice were perfused with PBS under anesthesia, and the left hemispheres were homogenized by sonication. To establish a standard curve, varying volumes of peripheral blood (0, 2, 4, 8, 16, and 32 µL) were added to homogenates of contralateral hemispheres, and the final volume was adjusted to 3 mL with PBS. For experimental samples, the left hemispheres were homogenized after MCAO, and hemoglobin concentration was determined using a hemoglobin assay kit (Sigma-Aldrich). For each sample, 0.4 mL homogenate was mixed with 1.6 mL reagent, and absorbance was measured at 540 nm with a spectrophotometer. Hemoglobin content was calculated

based on the standard curve.

BrdU Incorporation Assay in Neutrophils

To label proliferating neutrophils, mice received intraperitoneal injections of the thymidine analog bromodeoxyuridine (BrdU, 50 mg/kg; BD Biosciences). BrdU was administered immediately before MCAO induction and again at 24 h post-MCAO. BrdU incorporation was assessed using a BrdU Flow Kit (BD Biosciences) according to the manufacturer's instructions. Briefly, following surface antibody staining of neutrophils, cells were incubated on ice for 30 min with fixation/permeabilization buffer, washed, and further permeabilized. Cells were then refixed/permeabilized for 5 min on ice, treated with DNase to expose incorporated BrdU, and incubated with anti-BrdU antibody at room temperature for 20 min. BrdU expression in neutrophils was analyzed by a BD LSRFortessa flow cytometer.

Antibody-Mediated Cell Depletion and Blockade

For depletion of CD122 expressing cells, including CD8 Tregs, mice received intraperitoneal injections of anti-CD122 antibody (200 μg, clone TMβ-1, Bio X Cell) one day prior to MCAO induction. Control mice were injected with an equal amount of isotype control antibody.

Neutrophils and NK cells were depleted by intraperitoneal injection of anti-Ly6G (200 μg, clone 1A8, Bio X Cell) or anti-NK1.1 (200 μg, clone PK136, Bio X Cell) antibodies one day before MCAO. Control mice received isotype control antibodies.

For PD-L1 blockade, mice were administered by intravenous injection anti–PD-L1 antibody (100 μg, per day, clone B7-H1, Bio X Cell) immediately after MCAO induction and again at day 1 post-MCAO.

In Vivo Inhibitor Treatment

To inhibit glycolysis in vivo, mice received intraperitoneal injections of 2-deoxyglucose (2-DG; 0.5 g/kg, MCE) immediately after MCAO induction and again at day 1 and day 2 post-MCAO. Control mice received equal volumes of PBS.

In Vivo Labeling of Neutrophils

To track neutrophil clearance in vivo, mice received intravenous injections of PE-conjugated anti-Ly6G antibody (1A8, Biolegend, 127607) solution (containing 1.5 μg antibody) immediately after MCAO induction and again at day 1 post-MCAO.

In Vivo Neutrophil Clearance and Phagocytosis Assay

To assess neutrophil clearance and phagocytic activity in vivo, a pulse-labeling strategy was employed as previously described (3). Briefly, PE-conjugated anti-Ly6G antibody (1A8, Biolegend, 127607) was intravenously administered to label circulating neutrophils in vivo. As neutrophils underwent physiological clearance after MCAO, PE-labeled neutrophils were phagocytosed by monocytes/macrophages within clearance organs, including the bone marrow, spleen, and liver. Monocytes/macrophages that engulfed PE-labeled neutrophils subsequently acquired PE fluorescence, which was quantified by flow cytometry as an indicator of neutrophil phagocytic clearance. This assay therefore provides an in vivo functional readout of neutrophil clearance dynamics and phagocytic activity.

Flow Cytometry

Peripheral blood for flow cytometry was collected from anesthetized mice by cardiac puncture into EDTA-coated tubes. For assessment of transferred neutrophil survival, blood was sampled from the retro-orbital sinus at different time points. RBCs were lysed using RBC lysis buffer (Thermo Fisher), and leukocytes were pelleted by centrifugation to obtain single-cell suspensions. For lymphocyte analysis, equal volumes of PBS and blood were mixed and layered over an equal volume of Ficoll lymphocyte separation solution, followed by gradient centrifugation without braking. Mononuclear cells were collected from the interphase, washed, and resuspended to obtain single cell suspensions.

For analysis of brain-infiltrating immune cells, mice were deeply anesthetized and perfused intracardially with ice-cold PBS. The ischemic hemispheres were dissected

and dissociated using the Neural Tissue Dissociation Kit (T) (Miltenyi Biotec) and a gentleMACS™ Octo Dissociator with Heaters (Miltenyi Biotec) according to the manufacturer's instructions. The resulting cell suspension was filtered through a 70 μm cell strainer and centrifuged at 400 × g for 10 min. The pellet was resuspended in 10 mL of 30% Percoll solution and subjected to density gradient centrifugation (30%/70% Percoll, 800 × g, 30 min). Immune cells were obtained by aspirating the interphase layer, followed by washing with PBS and centrifugation to pellet the cells.

For bone marrow flow cytometry, femurs and tibias were collected after anesthesia, and bone marrow was flushed with PBS containing 5 mM EDTA and 1% FBS. The suspension was passed through a filter, treated with RBC lysis buffer, and centrifuged to collect bone marrow cells.

For preparation of liver and spleen, mice were anesthetized and the organs were harvested. Tissues were mechanically dissociated to obtain cell suspensions, which were filtered through a 70 μm cell strainer, centrifuged, and subsequently subjected to RBC lysis to yield single-cell suspensions.

For all samples, single-cell suspensions were incubated with Mouse BD Fc Block on ice for 10 min to block Fc receptors, followed by staining with appropriate fluorochrome-conjugated antibodies (1:200) for 30 min on ice. For intracellular staining, cells were processed using a fixation/permeabilization kit (Thermo Fisher) and incubated with intracellular antibodies. Cells were washed, resuspended in PBS, and analyzed using a flow cytometer. Flow cytometry data were analyzed with FlowJo (v10.6.2) software. Flow cytometry dimensionality-reduction visualizations were produced in Cytobank (Beckman Coulter).

FITC-anti-mouse-CD45 Antibody (30-F11, BD Biosciences, 553079), Pacific Blue™ anti-mouse CD45 Antibody (30-F11, Biolegend, 103126), APC anti-mouse CD45 Antibody (30-F11, Biolegend, 103112), APC anti-mouse CD45.1 Antibody (A20, Biolegend, 103112), PE/Cyanine7 anti-mouse/human CD11b Antibody (M1/70, Biolegend, 101216), PerCP/Cyanine5.5 anti-mouse Ly-6G Antibody (1A8, Biolegend, 127616), FITC anti-mouse Ly-6G Antibody (1A8, Biolegend, 127605), PE anti-mouse Ly-6G Antibody (1A8, Biolegend, 127607), PE anti-mouse CD19 Antibody (6D5,

Biolegend, 115508), FITC anti-mouse CD3e Antibody (145-2C11, BD Biosciences, 553061), BUV510 Anti-Mouse CD3 Antibody (145-2C11, BD Biosciences, 563024), APC-Cy7 Anti-Mouse CD4 Antibody(GK1.5, BD Biosciences, 552051), APC anti-mouse CD8a Antibody(53-6.7, BD Biosciences, 553035), PerCP/Cyanine5.5 anti-mouse CD8a Antibody (53-6.7, BioLegend, 100734), BV650 Anti-Mouse NK-1.1 Antibody (PK136, BD Biosciences, 564143), Brilliant Violet 421 anti-mouse/human KLRG1 (MAFA) Antibody (2F1/KLRG1 BioLegend, 138413), V500 anti-mouse CD44 Antibody (IM7, BD Biosciences, 560781), BUV395 Anti-Mouse CD62L Antibody (MEL-14, BD Biosciences, 740218), PE Anti-Mouse CD122 Antibody (TM-β1, BD Biosciences, 553362), BV421 Anti-mouse CD122 Antibody (5H4, BD Biosciences, 564925), BV605 anti-mouse Ly-49G2 Antibody (4D11, BD Biosciences, 742881), PE anti-mouse Ly-49F Antibody (H4F9, BD Biosciences, 550987), Brilliant Violet 650 anti-mouse CD274 (PD-L1) Antibody (10F.9G2, BioLegend, 124336), PE Anti-Hu/Mo HIF-1 alpha Antibody (Mgc3, BioLegend, 12-7528-82), CoraLite® Plus 488-conjugated Annexin A2 Polyclonal antibody (proteintech, CL488-11256), Brilliant Violet 605 anti-human CD62L (DREG-56, BioLegend, 304833), FITC anti-mouse Perforin (S16009A, BioLegend, 154309), Alexa Fluor® 647 anti-mouse EOMES (W17001A, BioLegend, 157703), Alexa Fluor 700 anti-mouse/human Helios Antibody (22F6, BioLegend, 137242)

APC anti-human CD16 (3G8, BioLegend, 302011), Brilliant Violet 421 anti-human CD274(PD-L1) (29E.2A3, BioLegend, 329713), FITC anti-human CD66b (G10F5, BioLegend, 305103), PE anti-human HIF1α (546-16, BioLegend, 359703), APC/Cyanine7 anti-human CD3 (UCHT1, BioLegend, 300425), Brilliant Violet 421 anti-human CD122(IL-2Rβ) (TU27, BioLegend, 339009), APC anti-huamn CD158b (DX27, BioLegend, 312611), FITC anti-human CD4 (A161A1, BioLegend, 357405), PerCP/Cyanine5.5 anti-human CD8 (SK1, BioLegend, 344709), FITC Anti-Human CD45(2D1, BD Biosciences, 572687), BUV395 Anti-Human CD31 (WM59, BD Biosciences, 565290)

Histological and Immunofluorescence Analysis

Mice were anesthetized and perfused intracardially with PBS followed by 4% paraformaldehyde (PFA). After fixation and dehydration, brains were cryosectioned. For immunofluorescence staining, 25 um brain sections from comparable anatomical levels were selected and permeabilized with PBS containing 0.5% Triton X-100 (PBST) for 15 min at room temperature and washed three times with 0.3% PBST (5 min each). Blocking was performed with 5% donkey serum in PBST for 1 h at room temperature. Sections were incubated overnight at 4 °C with the following primary antibodies: anti-Ly6G (Proteintech, 65140-1-Ig, 1:50), anti-CD31 (R&D Systems, AF3628, 1:200), and anti-ZO-1 (Abcam, ab221547, 1:100). The next day, sections were washed three times in 0.3% PBST (10 min each) and incubated with the corresponding secondary antibodies conjugated to Alexa Fluor 488, 555, 594, or 647 (Invitrogen, 1:500) for 1 h at room temperature in the dark. After three final washes in PBS, sections were mounted with DAPI-containing mounting medium (Abcam) and coverslipped.

For bone marrow staining, mouse hindlimbs were collected and fixed in 4% PFA overnight, decalcified in EDTA solution at 37 °C for approximately 2 weeks, dehydrated in sucrose, and embedded in OCT. Longitudinal frozen sections (30 μm) were prepared. Sections were permeabilized with 0.3% Triton X-100, blocked with 5% BSA, and incubated overnight at 4 °C with primary antibodies, including anti-Ly6G (Proteintech, 65140-1-Ig, 1:100), anti–PD-L1 (Abcam, ab213480, 1:100), and anti-CD68 (Invitrogen, PA5-78996, 1:100). After washing, sections were incubated with Alexa Fluor–conjugated secondary antibodies (1:500) and mounted with antifade medium containing DAPI.

Images were acquired using a Leica TCS SP8 laser scanning confocal microscope, and three random fields were captured for each sample. ImageJ (v1.54g) was employed to analyze the images.

For hematoxylin and eosin (HE) staining, mice were perfused intracardially with PBS followed by 4% PFA. Brains were embedded in paraffin and sectioned at 25 μm. HE staining was performed using standard protocols. Hemorrhagic foci were identified by the presence of extravasated erythrocytes, and hemorrhage area was quantified

using ImageJ (v1.54g).

Assessment of BBB Leakage

For endogenous IgG leakage, brain sections were blocked with 5% BSA for 1 h and then stained with FITC-conjugated donkey anti-mouse IgG (Invitrogen, 1:500). Leakage volume of endogenous IgG was calculated by integrating leakage areas across six serial sections at defined intervals.

For FITC–dextran leakage, mice were intravenously injected with 50 mg 4 kDa FITC–dextran 6 h before sacrifice, and brain tissues were subsequently sectioned and stained. Fluorescence microscopy was used to image each section, and the leakage area of endogenous IgG and FITC–dextran was quantified.

Behavioral Tests

The rotarod test and the adhesive removal test were used to assess neurological deficits after MCAO (4, 5). All behavioral experiments were performed in a double-blind manner, with investigators blinded to group allocation. Before MCAO surgery and behavioral assessments, mice were pre-trained for 3 consecutive days to adapt to the procedures. During the formal testing phase, each mouse was tested three times per day, and the mean value was used for analysis. The minimum interval between trials was 15 min.

Rotarod test

Mice were placed on an elevated rotating rod apparatus (Glord Tech Co. Ltd, Beijing, China), which forced them to walk forward. The rotation speed was set at 5 rpm initially and gradually increased to 65 rpm within 300 s. The latency to fall was recorded as a measure of motor coordination after MCAO.

Adhesive removal test

Small adhesive patches (2 × 3 mm) were gently placed on the forepaw contralateral to the ischemic hemisphere. The latency to contact and remove the patch was recorded. A maximum of 60 s was allowed for stimulus perception and 120 s for patch removal. This test was used to evaluate sensory and motor function after MCAO.

Neutrophil Isolation from Mice

Peripheral blood and femoral bone marrow were used for neutrophil isolation. Following anesthesia, peripheral blood was collected by cardiac puncture, and RBC were lysed to prepare single cell suspensions. Alternatively, one femur was dissected, surrounding soft tissue was removed, and the bone marrow cavity was flushed with HBSS to obtain single cell suspensions. Neutrophils were then isolated using a mouse neutrophil isolation kit (Miltenyi) according to the manufacturer's protocol. Briefly, cells were pelleted by centrifugation and resuspended in 200 μL PBS containing FBS, followed by incubation with 50 μL biotin-conjugated antibody mix for 10 min on ice. After washing and resuspension in PBS, cells were incubated with magnetic microbeads for 15 min on ice and subsequently applied to magnetic columns. The enriched neutrophil fraction was collected in fresh tubes. Isolated neutrophils were used for RNA sequencing and functional assays. Alternatively, neutrophils from CD45.1 mice were intravenously transferred into WT mice to monitor their survival. In some experiments, neutrophils cultured in vitro were stimulated with LPS.

Giemsa staining

Peripheral neutrophils were isolated from mouse blood by RBC lysis, cytospun onto glass slides, fixed in methanol, and stained with Giemsa (1:20 in pH 6.8 buffer) for 12 min at room temperature. Slides were rinsed in pH 6.8 buffer, air-dried, and mounted. Nuclear morphology was imaged under a bright-field microscope (Leica).

Flow Cytometric Sorting of Mouse Cells

Single-cell suspensions were prepared from spleens and lymph nodes (cervical, axillary, and inguinal) of mice. Briefly, tissues were dissected after anesthesia and mechanically dissociated through gentle grinding. Cells were then subjected to Ficoll density gradient centrifugation to obtain mononuclear cells from the interphase. For surface staining, cells were incubated on ice for 30 min with the following antibodies: FITC anti-mouse CD3e Antibody (145-2C11, BD Biosciences, 553061), PE anti-mouse CD19 Antibody (6D5, Biolegend, 115508), APC-Cy7 Anti-Mouse CD4 Antibody(GK1.5, BD

Biosciences, 552051), APC anti-mouse CD8a Antibody(53-6.7, BD Biosciences, 553035), BV421 Anti-mouse CD122 Antibody (5H4, BD Biosciences, 564925), PE anti-mouse Ly-49F Antibody (H4F9, BD Biosciences, 550987), BV650 Anti-Mouse NK-1.1 Antibody (PK136, BD Biosciences, 564143), and Brilliant Violet 421 anti-mouse/human KLRG1 (MAFA) Antibody (2F1/KLRG1 BioLegend, 138413). Flow cytometric sorting was performed on a BD FACSAria III cell sorter. Populations were defined as follows: T cells ($CD3^+$), B cells ($CD19^+$), CD8 Treg cells ($CD3^+CD8^+CD122^+Ly49F^+$), NKT cells ($CD3^+NK1.1^+$), and CD8 EF cells ($CD3^+CD8^+KLRG1^+$).

For adoptive transfer experiments, sorted cells were administered via tail vein injection immediately after MCAO surgery. Typically, 2×10^6 sorted T or B cells, or 0.5×10^5 sorted CD8 Treg, NKT, or CD8 EF cells were transferred into recipient mice. Control mice received an equal volume of PBS. For in vitro culture, sorted cells were stimulated with Dynabeads Mouse T-Activator CD3/CD28 (Thermo Fisher) following the manufacturer's instructions, and maintained in medium supplemented with IL-2 (5 ng/mL, BioLegend) and IL-15 (5 ng/mL, BioLegend).

Cell Culture

Neutrophils and CD8 Tregs were cultured in RPMI 1640 medium (Gibco) supplemented with 10% FBS (VivaCell) and 1% penicillin–streptomycin (Gibco), IL-2, and IL-15. Freshly isolated neutrophils, as well as neutrophils cultured for 24 h and 48 h, were subjected to flow cytometry, immunofluorescence staining and RNA-seq to characterize time-dependent changes.

An indirect co-culture system was established using Transwell inserts with 0.4 μm pore size and 11 mm diameter (Corning). Neutrophils (5×10^5 cells) were cultured in the lower chamber, while CD8 Tregs (2.5×10^5 cells) were seeded in the upper chamber, corresponding to an approximate CD8 Treg:neutrophil ratio of 1:2. Culture medium was replaced with fresh medium at the start of co-culture, and medium was refreshed as required during the experiments. Neutrophils cultured alone or after co-culture were subsequently subjected to flow cytometry, immunofluorescence staining, endothelial

permeability assays, metabolic assays, and RNA-seq. For flow cytometry, cytokine profiling, RNA-seq, and metabolic assays, neutrophils were analyzed at 24 hours after the initiation of co-culture. For apoptosis assays, neutrophils were assessed at 6 hours after co-culture initiation. In all experiments, neutrophils cultured alone for equivalent durations served as controls.

Cytokine Array Assay

Neutrophils cultured alone or co-cultured with CD8 Tregs for 24 h after LPS stimulation were lysed in RIPA buffer (Millipore), and protein concentrations were quantified. For the cytokine array (R&D Systems, ARY028), membranes were first blocked in Array Buffer 6, followed by incubation with diluted protein samples overnight at 2–8 °C on a rocking platform. After washing, membranes were sequentially incubated with a detection antibody cocktail, streptavidin-HRP, and chemiluminescent substrate. Signals were detected by iBright FL1500 Imaging System.

Flow Cytometric Analysis of Neutrophil and CD8 Treg Apoptosis

Peripheral blood cells, including CD8 Tregs and neutrophils, as well as neutrophils from in vitro culture conditions, were assessed for apoptosis using an Annexin V-FITC/propidium iodide (PI) apoptosis detection kit (BioLegend) according to the manufacturer's instructions. Data acquisition was performed on a BD LSRFortessa flow cytometer, and analysis was conducted using FlowJo (v10.6.2) software.

In Vitro BBB and OGD Models, FITC–Dextran Permeability, and Neutrophil Transmigration Assays

An in vitro BBB model was established as previously described (6). Transwell co-culture inserts with polyethylene terephthalate (PET) membranes (0.4 µm or 3 µm pore size, 11 mm diameter; Corning) were coated with collagen and fibronectin. For mouse in vitro experiments, bEnd.3 cells were seeded at a density of 1 × 10^5 cells per insert and cultured at 37 °C with 5% CO_2 until a confluent monolayer was formed. For human in vitro experiments, brain microvascular endothelial cells isolated from resected

temporal lobe tissue were seeded at the same density (1 × 10^5 cells per insert). Transendothelial electrical resistance (TEER) measurement was used to confirm the formation of tight junctions in endothelial monolayers. Depending on the experimental purpose, neutrophils (5 × 10^5 cells) and/or CD8 Tregs (2.5 × 10^5 cells) were added to the upper chamber of the transwell system to assess their effects on endothelial barrier permeability. The OGD model was established by replacing the culture medium with glucose-free, serum-free medium, followed by incubation of endothelial cells under hypoxic conditions for 4 h. The medium was then replaced with glucose-containing medium supplemented with 10% FBS, and cells were maintained under normoxic conditions (95% air, 5% CO_2) for recovery.

To evaluate BBB permeability after OGD, 4 kDa FITC–dextran (Sigma-Aldrich) was added to the upper chamber, and fluorescence intensity in the lower chamber was measured every 1 hour using a microplate reader (SpectraMax M5, Molecular Devices). FITC–dextran concentration was calculated based on a standard curve generated from known concentrations.

For neutrophil transmigration assays, Transwell inserts with a 3 μm pore size and 11 mm diameter (Corning) were used. bEnd.3 cells or primary human brain microvascular endothelial cells were seeded on the upper membrane and subjected to OGD pretreatment. The lower wells were coated with PLL to facilitate adherence of transmigrated neutrophils. Neutrophils from mice or patients, cultured alone or indirectly co-cultured with CD8 Tregs for 24 h, were added to the upper chamber. The number of neutrophils that migrated to the lower chamber was examined under a microscope at defined time points.

In Vitro Immunofluorescence Staining

For in vitro staining, neutrophils and endothelial cells were seeded on PLL-coated coverslips and fixed with 4% PFA for 30 min, followed by blocking with 5% BSA for 1 h at room temperature. Cells were then incubated overnight at 4 °C with primary antibodies: anti-CD31 (R&D Systems, AF3628, 1:200) and anti–ZO-1 (Abcam, ab221547, 1:200) for endothelial cells; anti-GLUT1 (1:200) for mouse neutrophils;

and anti–PD-L1 (1:100) and anti–HIF-1α (1:100) for human neutrophils. The following day, cells were washed with PBS and incubated with the appropriate fluorophore-conjugated secondary antibodies. Coverslips were mounted with antifade medium, and images were acquired using a Leica TCS SP8 laser scanning confocal microscope.

Seahorse Assays

PLL-coated culture plates were used to seed neutrophils (2×10^5 per well) that had been cultured alone or indirectly co-cultured with CD8 Tregs. The Glycolysis Stress Test Kit (Agilent 103017-100) was applied according to the manufacturer's instructions, and ECAR was measured using the Agilent Seahorse XFp Analyzer. During the assay, neutrophils were sequentially treated with glucose (10 mM), oligomycin (1.0 mM), and 2-deoxyglucose (2-DG, 50 mM).

Metabolite Extraction and HPIC-MRM-MS Analysis

Metabolites were extracted from samples using pre-cooled MeOH/H_2O (3:1), followed by freeze–thaw cycles, sonication, centrifugation, drying, and reconstitution. Standard calibration solutions were prepared from stock standards by serial dilution. Metabolite separation was performed on a Thermo Dionex ICS-6000 HPIC system, and detection was carried out using an AB SCIEX 6500 QTRAP+ triple quadrupole mass spectrometer in MRM mode. Calibration curves were generated with 1/x weighting, and limits of detection (LOD) and quantitation (LOQ) were determined based on signal-to-noise ratios of 3 and 10, respectively. The final concentration was obtained by multiplying the calculated concentration by the dilution factor. The metabolite concentration per 10^7 cells was calculated as final concentration × final volume ÷ total cell number. Differential metabolite analysis was performed using Student's t test, and Variable importance in projection (VIP) scores were calculated from the orthogonal partial least squares discriminant analysis (OPLS-DA) model for each pairwise group comparison. Metabolites with VIP > 1 and $p < 0.05$ were considered statistically significant. Metabolite set enrichment analysis was conducted using MetaboAnalyst (7).

Bulk RNA Sequencing

Total RNA was extracted from isolated neutrophils using TRIzol, and samples with high integrity (RIN > 8.8) were used for library construction. Poly(A)+ mRNA was enriched, fragmented, and reverse-transcribed to generate cDNA. After end repair, adaptor ligation, and PCR amplification, libraries were quality-checked and sequenced on the Illumina NovaSeq 6000 platform with paired-end 150 bp reads (PE150).

Single-cell RNA Sequencing

Peripheral immune cells were isolated from mouse peripheral blood collected via cardiac puncture under anesthesia. RBCs were removed by RBC lysis followed by repeated washing. The remaining immune cells were resuspended in cold PBS to prepare a single-cell suspension. Library preparation and sequencing were performed by Novogene. Briefly, single-cell capture and barcoding were carried out using the Chromium Single Cell Controller (10x Genomics), and libraries were constructed following the manufacturer's protocol. Sequencing was performed on the Illumina NovaSeq 6000 platform.

RNA-seq Data Analysis

Raw sequencing reads were aligned to the mouse reference genome (mm10) using HISAT2 (v2.2.1), and gene-level count matrices were generated. Differential expression analysis was performed using DESeq2 (v1.46.0) and edgeR (v4.4.2), based on raw counts. PCA was used for sample clustering. Functional enrichment of differentially expressed genes was conducted using Metascape. Heatmaps and other visualizations were generated using the R packages pheatmap (v1.0.12) and ggplot2 (v3.5.1). Time-series clustering of gene expression profiles was performed using the Mfuzz (v2.66.0).

Single-cell RNA-seq Data Analysis

Raw sequencing data were quality-checked using FastQC. Gene expression matrices were generated by aligning reads to the mouse reference genome (mm10) using Cell

Ranger (v6.0.0, 10x Genomics). Downstream analysis was performed in R (v4.4.3) using Seurat (v4.4.0) and Harmony (v1.2.3). Cells expressing fewer than 200 or with >10% mitochondrial gene content were excluded. Gene expression matrices were log-normalized, and the top 2000 highly variable genes were used for PCA. Cell clustering was performed using FindNeighbors and FindClusters. Differentially expressed genes (DEGs) between clusters were identified using the Wilcoxon rank-sum test (adjusted by Bonferroni correction) via FindMarkers and FindAllMarkers (see **Table S2** *for the full DEG list). Module scores for biological processes, including neutrophil aging signatures, were calculated using AddModuleScore based on previously published gene sets (***Table S4***). Visualization was performed using ggplot2 (v3.5.1).*

Cell–cell communication and pseudotime analysis

To investigate intercellular communication between neutrophils and other immune subsets, we used CellChat (8) (v1.6.1) to infer ligand–receptor interactions. The expression matrix was normalized and preprocessed using the default pipeline, and signaling interactions were computed using the computeCommunProb and computeCommunProbPathway functions. Significant signaling pathways were ranked based on communication probability and information flow.

To assess the differentiation trajectories of neutrophils, we applied scTour (9) (v1.0.0) to infer latent developmental states. To validate the inferred trajectory, we independently performed pseudotime analysis using monocle2 (10) (v2.34.0), monocle3 (11) (v1.3.1), and CytoTRACE2 (12) (v1.0.0). For monocle2, ordering was based on highly variable genes calculated in Seurat, while in monocle3, trajectory graphs were constructed with learn graph and pseudotime was calculated via order_cells. For CytoTRACE2, we used the raw counts matrix to compute differentiation potential based on gene diversity and expression entropy.

Statistical Analysis

All data are presented as mean ± standard deviation (SD) or mean ± standard error

(SEM). Statistical analyses of single-cell RNA-seq data were performed using the Wilcoxon rank sum test. Comparisons between two groups with normal distribution and equal variance were conducted using 2-tailed Student's t tests. One-way ANOVA was used for comparing means among multiple groups. For repeated measures over time, differences were assessed using two-way repeated-measures ANOVA or a mixed-effects model where appropriate. All statistical analyses were performed using R (version 4.4.3) or GraphPad Prism (version 10.4.0). A p-value less than 0.05 was considered statistically significant. Data quantification was conducted independently by two blinded observers.

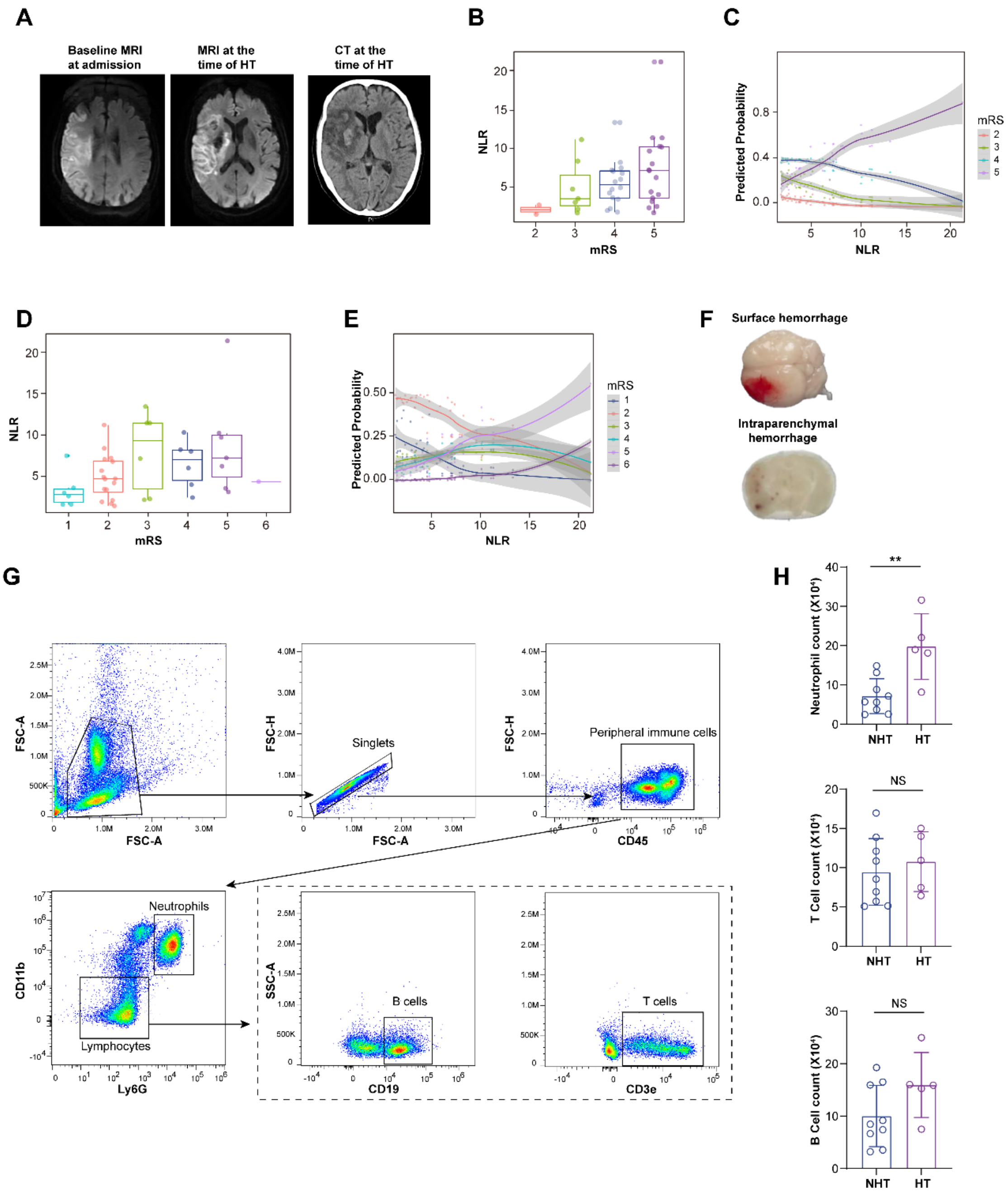

Figure S1. Relationship between NLR and neurological recovery, related to Figure 1.

(A) *Representative MRI images from ischemic stroke patients and CT scans demonstrating HT.*

(B) *NLR values in stroke patients stratified by mRS scores at discharge.*

(C) *Predictive value of NLR for discharge mRS outcomes.*

(D) *NLR values in stroke patients stratified by mRS scores at 3 months.*

(E) *Predictive value of NLR for 3-month mRS outcomes.*

(F) *Representative images of HT in mouse brains at day 3 after MCAO.*

(G) *Gating strategy for flow cytometric identification of neutrophils, T cells, and B cells in mouse peripheral blood.*

(H) *Comparison of neutrophil, T cell, and B cell counts between HT (n = 5) and NHT (n = 9) mice at day 3 post-MCAO.*

*Data are presented as mean ± SD. NS, not significant; **p < 0.01. Student's t test (H).*

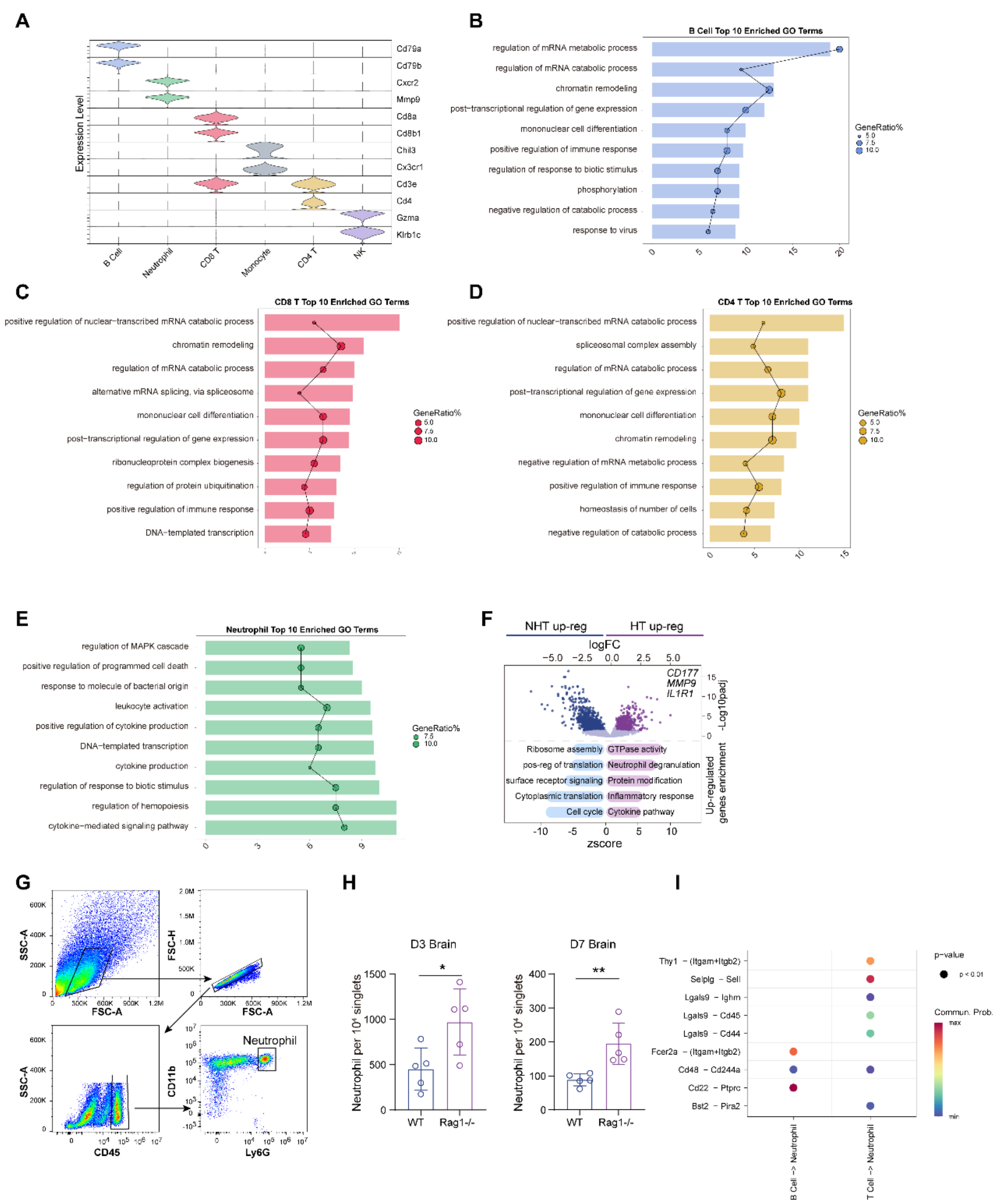

Figure S2. Functional alterations of peripheral immune cells in HT mice, related to Figure 1.

(A) *Violin plots showing expression of marker genes in peripheral immune cells from mice.*

(B–E) *Top 10 enriched GO pathways of upregulated genes in B cells (B), $CD8^+$ T cells (C), $CD4^+$ T cells (D), and neutrophils (E) from HT compared with NHT mice.*

(F) *Differential gene expression analysis of neutrophils between HT and NHT patients. Volcano plot (top) depicts log fold change (logFC) versus adjusted p-value. Pathway enrichment analysis (bottom) shows significantly enriched pathways, with the x-axis indicating z-scores.*

(G) *Gating strategy for flow cytometric identification of brain-infiltrating neutrophils.*

(H) *Quantification of brain-infiltrating neutrophils in WT and Rag1-/- mice at day 3 (n = 5 per group) and day 7 (n = 5 per group) post-MCAO.*

(I) *Cell–cell interaction analysis showing ligand–receptor pairs mediating regulatory effects of B cells and T cells on neutrophils in peripheral blood.*

*Data are presented as mean ± SD. *$p < 0.05$, **$p < 0.01$. Student's t test (H).*

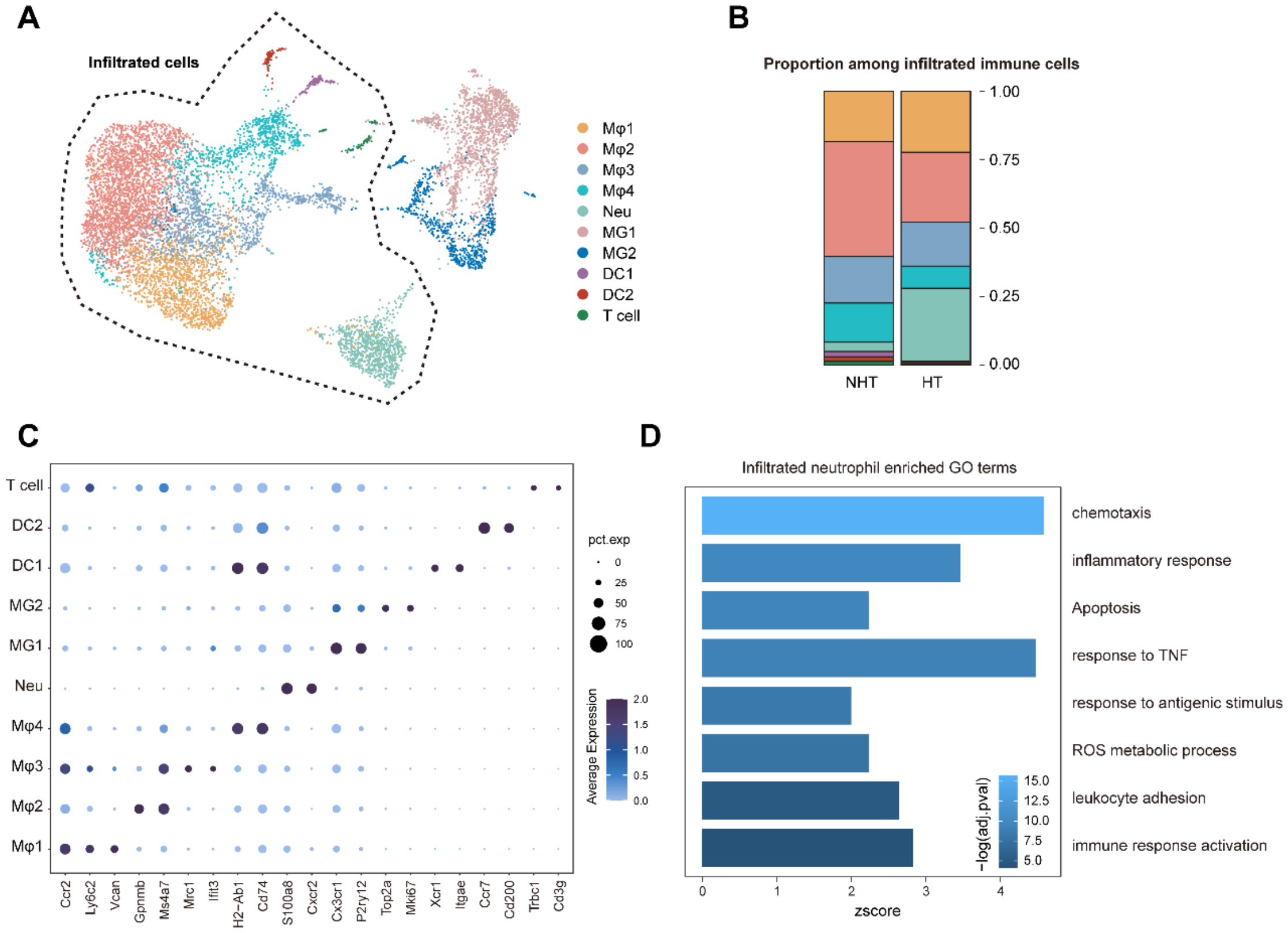

Figure S3. scRNA-seq analysis of brain-infiltrating immune cells, related to Figure 1.

(A) *UMAP visualization of brain-infiltrating immune cell clusters from NHT and HT mice.*

(B) *Bar plots showing the proportional composition of brain-infiltrating immune cell populations in NHT and HT mice.*

(C) *Dot plot showing the expression of canonical marker genes across brain-infiltrating immune cell clusters.*

(D) *Bar plot showing the top enriched GO biological process pathways of upregulated genes in brain-infiltrating neutrophils from HT compared with NHT mice.*

Mφ, macrophage; Neu, neutrophil; MG, microglia; DC, dendritic cell.

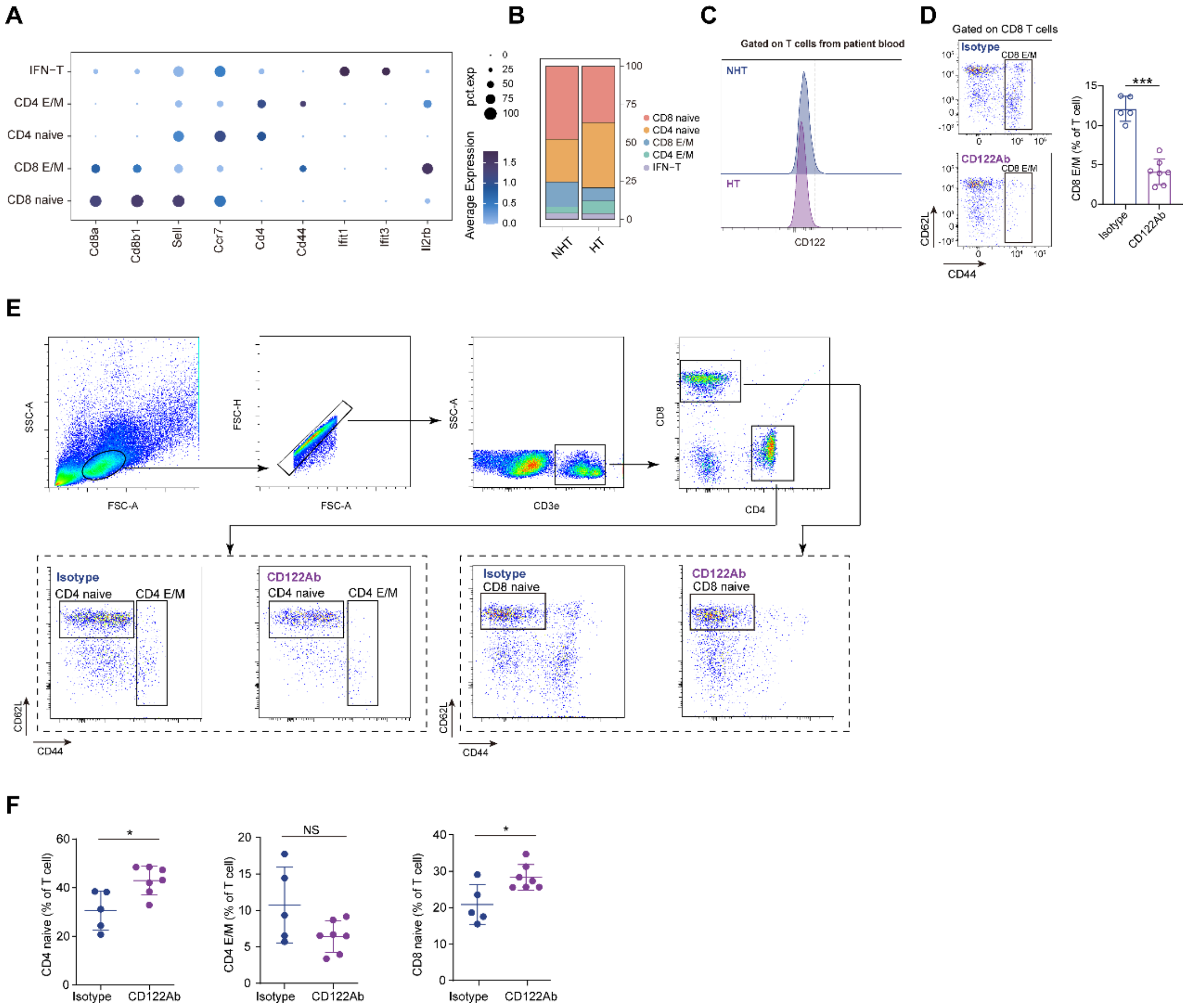

Figure S4. Effects of CD122Ab on peripheral T cell subsets, related to Figure 2.

(A) *Dot plot showing marker genes of peripheral T cell subsets in mice.*

(B) *Comparison of proportions of T cell subsets between HT and NHT mice.*

(C) *Representative flow cytometry plots showing the proportion of CD122*^{hi} *T cells in peripheral blood from HT and NHT patients.*

(D) *Flow cytometry plots showing CD8 E/M cells (left) and quantification of their proportions within total T cells in isotype control (n = 5) and CD122Ab-treated (n = 7) mice (right).*

(E) *Gating strategy for peripheral CD4 naïve, CD4 E/M, and CD8 naïve T cells in mice.*

(F) *Quantification of CD4 naïve, CD4 E/M, and CD8 naïve T cells in isotype (n = 5) and CD122Ab (n = 7) treated mice.*

*Data are presented as mean ± SD. NS, not significant; *p < 0.05, ***p < 0.001. Student's t test (D, F).*

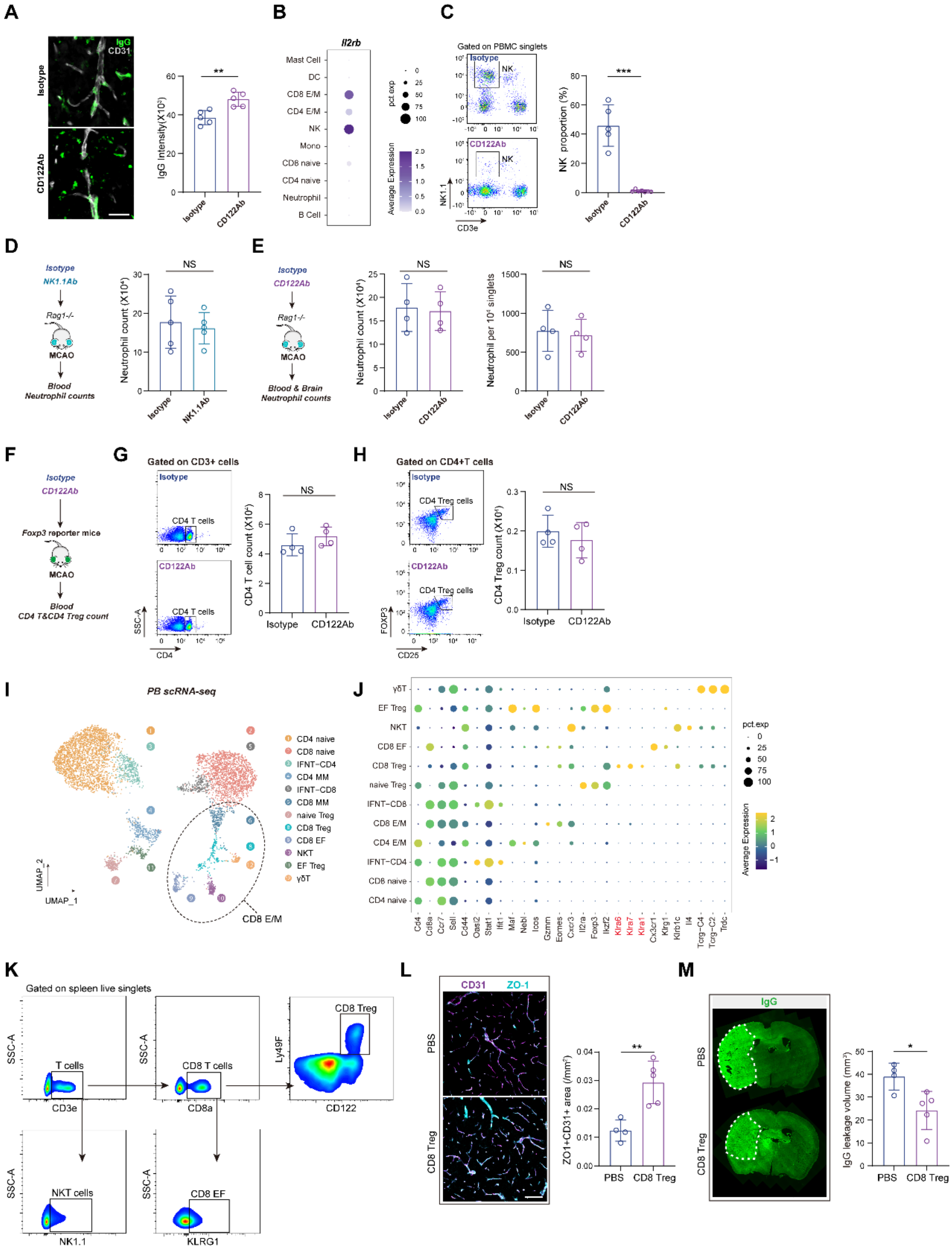

Figure S5. CD122Ab exacerbates BBB disruption after MCAO primarily through effects on T cells, related to Figure 2.

(A) *Representative immunofluorescence images of CD31 and IgG staining in brain sections (left) and quantification of IgG intensity (right) in isotype and CD122Ab treated mice (n = 5 per group; scale bar, 50 μm).*

(B) *Dot plot showing Il2rb expression across peripheral immune cell populations.*

(C) *Flow cytometry plots showing NK cells (left) and quantification of their proportions within CD3□ cells in isotype control (n = 5) and CD122Ab-treated (n = 7) mice (right).*

(D) *Schematic illustration of experimental workflow for isotype and NK1.1Ab treated Rag1-/- mice (left), and quantification of peripheral neutrophils (n = 5 per group).*

(E) *Schematic illustration of experimental workflow for isotype and CD122Ab treated Rag1-/- mice (left), and quantification of peripheral (middle) and brain-infiltrating (right) neutrophils (n = 4 per group).*

(F) *Schematic illustration of the experimental workflow for assessing the effects of CD122Ab treatment on CD4 T cells and CD4 Tregs using Foxp3 reporter mice.*

(G) *Representative flow cytometry plots showing CD4 T cells in peripheral blood of isotype control and CD122Ab-treated mice (left), and quantification of CD4 T cell numbers between groups (right) (n = 4 per group).*

(H) *Representative flow cytometry plots showing CD4 Tregs (FOXP3-GFP^{+}) in peripheral blood of isotype control and CD122Ab-treated mice (left), and quantification of CD4 Treg numbers between groups (right) (n = 4 per group).*

(I) *UMAP visualization of peripheral T cell subsets in mice.*

(J) *Dot plot showing marker genes of peripheral T cell subsets.*

(K) *Flow cytometric gating strategy for sorting CD8 Tregs, NKT cells, and CD8 EF cells.*

(L) *Representative immunofluorescence images of CD31 and ZO-1 staining in brain sections from mice receiving PBS or CD8 Treg adoptive transfer (left; scale bar, 100 μm), and quantification of ZO-1^{+} endothelial coverage between groups (right; PBS, n = 4; CD8 Treg, n = 5).*

(M) *Representative immunofluorescence images of endogenous IgG staining in brain sections from mice receiving PBS or CD8 Treg adoptive transfer (left), and quantification of IgG leakage volume between groups (right; PBS, n = 4; CD8 Treg, n = 5).*

*Data are presented as mean ± SD. NS, not significant; *p < 0.05, **p < 0.01, ***p < 0.001. Student's t test (A, C, D, E, G, H, L, M).*

IFNT-CD4, IFN-responsive CD4 T cells; IFNT-CD8, IFN-responsive CD8 T cells;

CD4 MM, CD4 memory T cells; CD8 MM, CD8 memory T cells; CD8 EF, CD8 effector T cells; EF Treg, CD4 effector regulatory T cells.

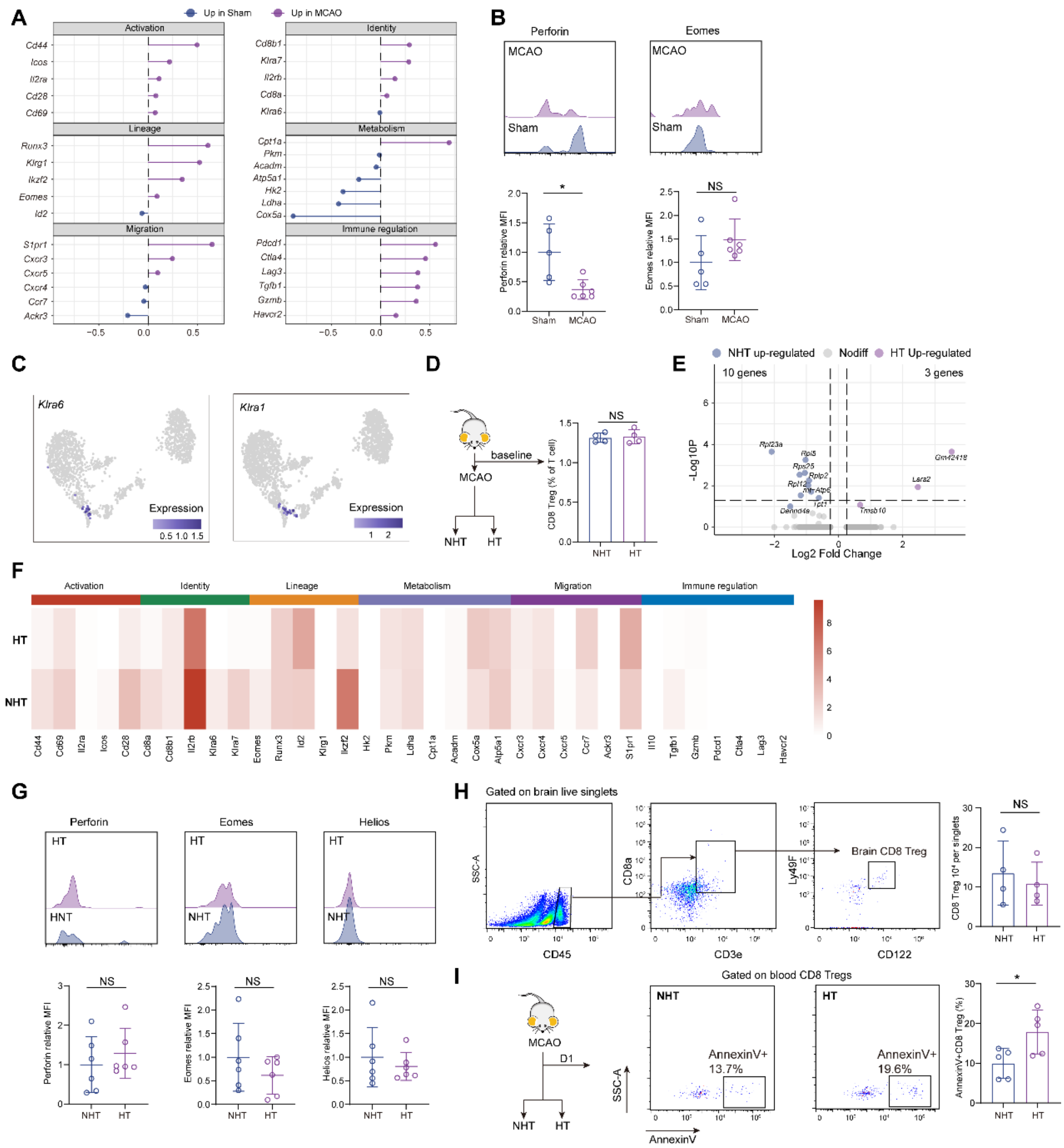

Figure S6. Characterization of CD8 Tregs following stroke and in the context of HT, related to Figure 3.

(A) *Lollipop plot showing expression differences (MCAO − Sham) of CD8 Treg functional genes based on bulk RNA-seq data.*

(B) *Representative flow cytometry plots showing Perforin and Eomes expression in peripheral CD8 Tregs from sham and MCAO mice at day 1 post-MCAO (top), and quantification of relative Perforin and Eomes MFI normalized to the sham group (bottom; sham, n = 5; MCAO, n = 6).*

(C) *Feature plots showing the expression of CD8 Treg marker genes Klra6 and Klra1 across peripheral T cell clusters.*

(D) *Schematic illustration of the experimental workflow for assessing baseline peripheral CD8 Treg numbers in HT and NHT mice, with HT and NHT groups retrospectively stratified based on hemorrhage status at day 3 post-MCAO (left), and comparison of baseline CD8 Treg proportions within total T cells between the two groups (right; n = 4 per group).*

(E) *Volcano plot showing differentially expressed genes between CD8 Tregs from HT and NHT mice. The x-axis represents log_2 fold change and the y-axis represents $-log_{10}$ adjusted p-value. Differentially expressed genes were defined as adjusted $p < 0.1$.*

(F) *Heatmap showing the expression of representative genes across functional categories, including activation, immune suppression, lineage stability, migration, and metabolic programming, in CD8 Tregs from NHT and HT mice.*

(G) *Representative flow cytometry plots showing Perforin, Eomes, and Helios expression in peripheral CD8 Tregs from NHT and HT mice (top), and quantification of relative Perforin, Eomes, and Helios MFI normalized to the NHT group (bottom; n = 6 per group).*

(H) *Gating strategy for flow cytometric identification of brain-infiltrating CD8 Tregs (left), and quantification of brain-infiltrating CD8 Treg numbers between NHT and HT mice (right) (n = 4 per group).*

(I) *Schematic illustration of the experimental workflow for assessing CD8 Treg apoptosis at day 1 post-MCAO in HT and NHT mice, with HT and NHT groups retrospectively stratified based on hemorrhage status at day 3 post-MCAO (left). Representative flow cytometry plots showing Annexin V^+ CD8 Tregs in peripheral blood from NHT and HT mice at day 1 post-MCAO (middle), and quantification of the proportion of Annexin V^+ cells within CD8 Tregs between the two groups (right; n = 5 per group).*

*Data are presented as mean ± SD. NS, not significant; *$p < 0.05$. Student's t test (B, D, G-I).*

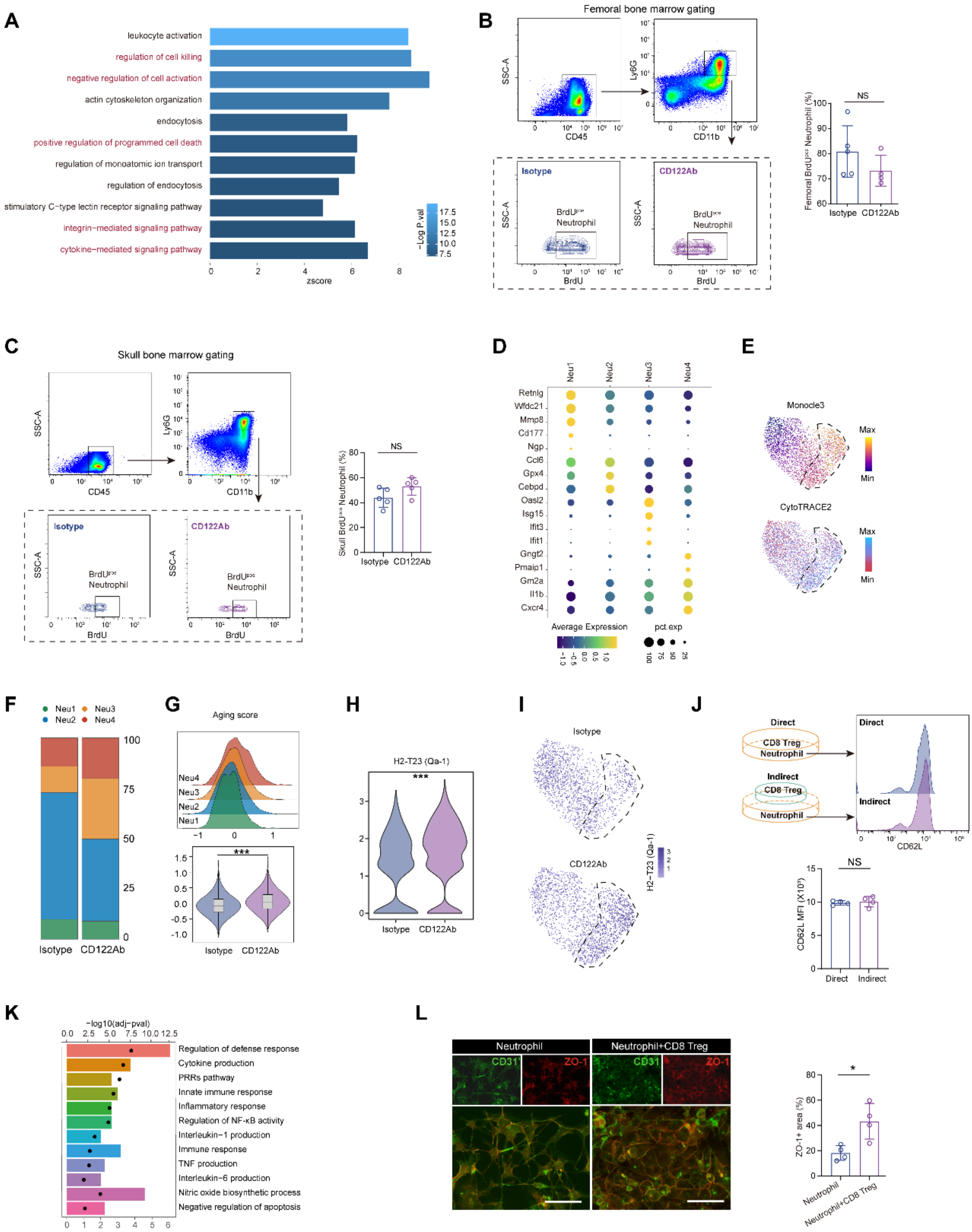

Figure S7. CD122Ab extends peripheral neutrophil lifespan, related to Figure 3 and Figure 4.

(A) *GO functional enrichment analysis of genes upregulated in CD8 Tregs compared with other T cell subsets based on scRNA-seq data.*

(B) *Gating strategy for BrdU^{pos} neutrophils in bone marrow (left), and comparison of BrdU^{pos} neutrophil proportions between isotype (n = 5) and CD122Ab (n =4) treated*

mice (right).

(C) *Gating strategy for BrdU^{pos} neutrophils in skull bone marrow (left), and comparison of BrdU^{pos} neutrophil proportions between isotype control and CD122Ab-treated mice (right) (n = 5 per group).*

(D) *Dot plot showing marker genes of peripheral neutrophil subclusters.*

(E) *Estimated neutrophil differentiation states inferred by Monocle3 and CytoTRACE2.*

(F) *Comparison of proportions of peripheral neutrophil subclusters between isotype and CD122Ab treated mice.*

(G) *Ridge plots showing aging score distribution across peripheral neutrophil subclusters (top), and comparison of aging scores between isotype and CD122Ab treated neutrophils (bottom).*

(H) *Violin plots showing the expression of H2-T23 in peripheral neutrophils from isotype control and CD122Ab-treated mice based on scRNA-seq data.*

(I) *Feature plots showing the distribution of H2-T23 expression in peripheral neutrophils from isotype control and CD122Ab-treated mice based on scRNA-seq data.*

(J) *Schematic illustration of the experimental workflow for direct and indirect (transwell) co-culture of CD8 Tregs and neutrophils. Representative flow cytometric histogram overlays showing CD62L expression on neutrophils under direct and indirect co-culture conditions (top), and quantification of CD62L MFI between groups (bottom) (n = 4 per group).*

(K) *Functional enrichment of genes upregulated in CD122Ab treated neutrophils compared with isotype group in bulk RNA-seq analysis. Bars represent adjusted p-values, and dots indicate z-scores.*

(L) *Representative immunofluorescence images of endothelial ZO-1 and CD31 staining following OGD (left) and quantification of ZO-1 coverage area (right; n = 4 per group; scale bar, 50 μm).*

*Data are presented as mean ± SD. NS, not significant; *$p < 0.05$, ***$p < 0.001$. Student's t test (B, C, J, L). Bonferroni-corrected Wilcoxon rank-sum test (G, H).*

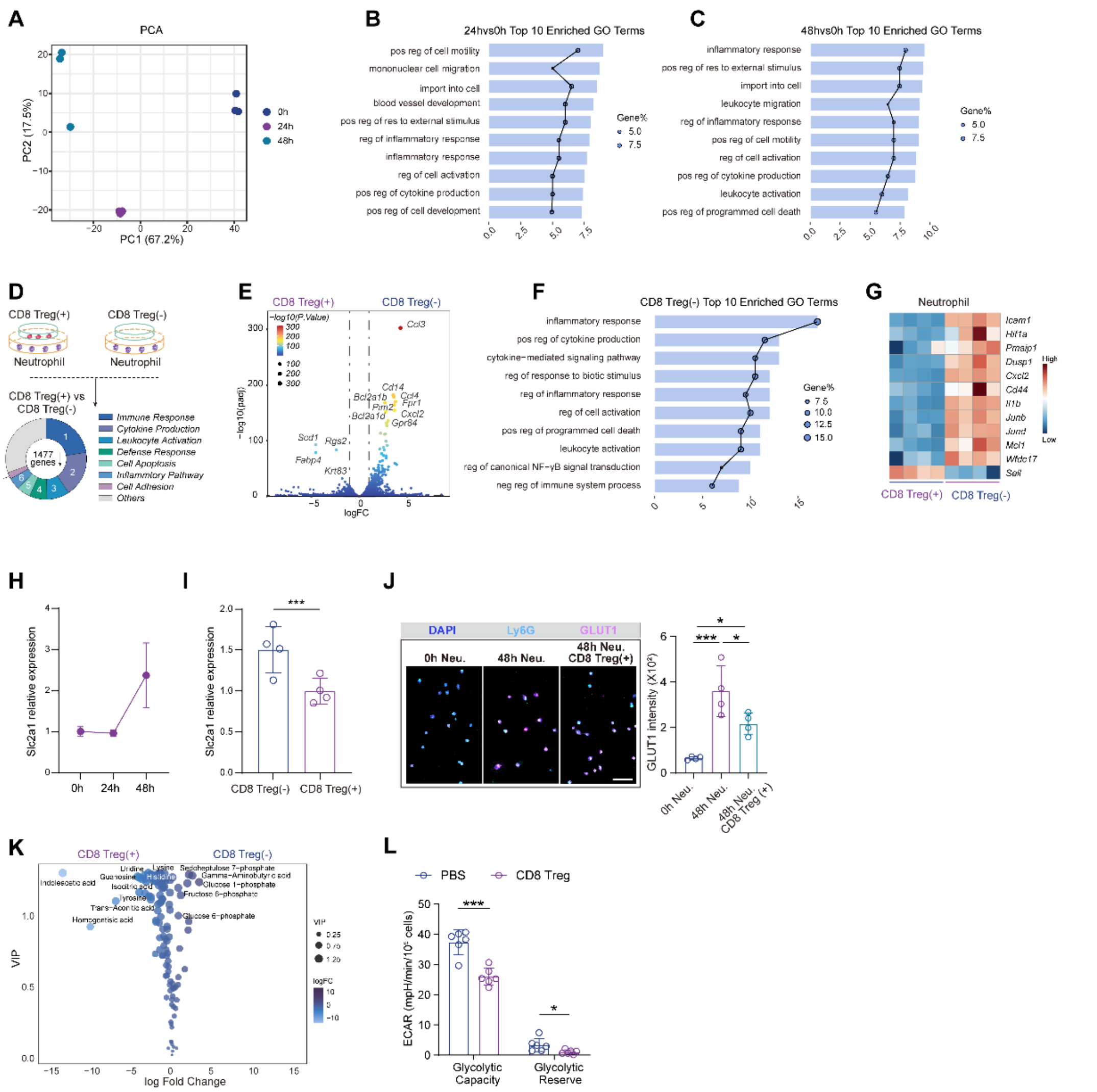

Figure S8. Long-term culture and co-culture models reveal metabolic and PD-L1 alterations in neutrophils, related to Figure 5.

(A) *PCA of transcriptomic profiles of neutrophils at 0 h, 24 h, and 48 h.*

(B) *GO enrichment pathways of genes upregulated at 24 h compared with 0 h.*

(C) *GO enrichment pathways of genes upregulated at 48 h compared with 0 h.*

(D) *Classification and proportions of enriched pathways for downregulated genes in neutrophils co-cultured with CD8 Tregs compared with neutrophils cultured alone.*

(E) *Volcano plot showing differentially expressed genes between neutrophils cultured alone and those co-cultured with CD8 Tregs.*

(F) *GO enrichment pathways of genes upregulated in neutrophils cultured alone compared with those co-cultured with CD8 Tregs.*

(G) *Heatmap showing expression of long-lived neutrophil–associated genes in*

neutrophils cultured alone versus co-cultured with CD8 Tregs.

(H) *Relative Slc2a1 expression in neutrophils from the long-term culture model, normalized to the 0-h group (bulk RNA-seq data; n = 3 per time point).*

(I) *Relative Slc2a1 expression in neutrophils cultured alone versus co-cultured with CD8 Tregs, normalized to the co-culture group (data from bulk RNA-seq, n = 4).*

(J) *Representative immunofluorescence images of Ly6G and GLUT1 staining (left) and quantification of GLUT1 mean fluorescence intensity (MFI; right) in each group (n = 4 per group; scale bar, 50 μm).*

(K) *Volcano plot showing differentially abundant metabolites between neutrophils cultured alone and those co-cultured with CD8 Tregs.*

(L) *Glycolytic capacity and glycolytic reserve in neutrophils cultured alone versus co-cultured with CD8 Tregs (n = 6 per group).*

*Data are presented as mean ± SD. *p < 0.05, ***p < 0.001; Student's t test (L), one-way ANOVA with Tukey's test (J), DESeq2 differential expression analysis (I).*

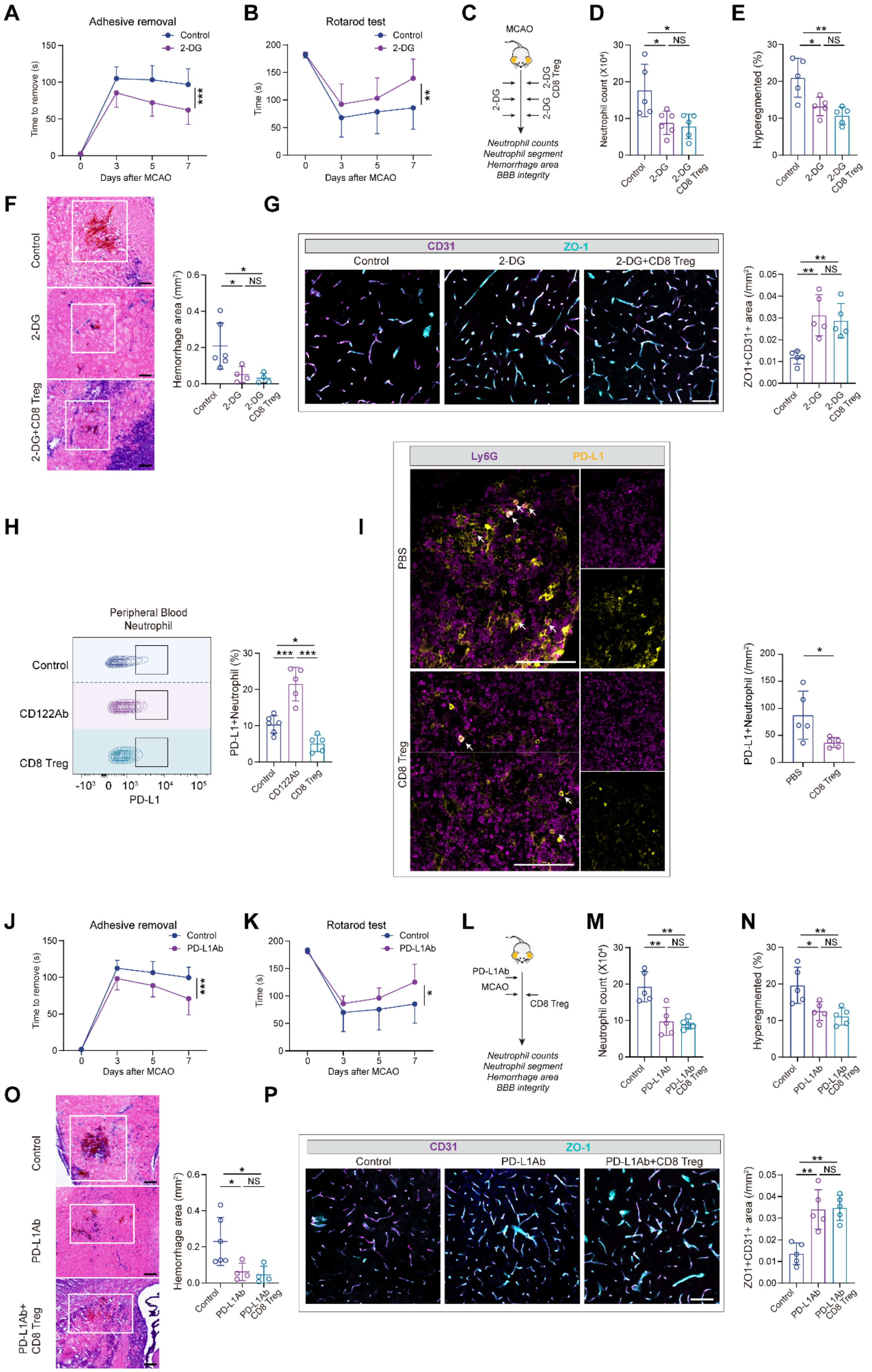

Figure S9. Role of glycolysis and PD-L1 in CD8 Treg–mediated regulation, related to Figure 5.

(A-B) *Neurological recovery of 2-DG-treated and control mice after MCAO, assessed by the adhesive removal test (A) and rotarod test (B) (n = 8 per group).*

(C) *Schematic illustration of the experimental workflow for 2-DG treatment in MCAO mice.*

(D) *Quantification of peripheral neutrophil counts in control, 2-DG, and 2-DG + CD8 Treg groups (control and 2-DG + CD8 Treg groups, n = 5; 2-DG group, n = 6).*

(E) *Quantification of the proportion of hypersegmented neutrophils across groups (n = 5 per group).*

(F) *Representative HE-stained coronal brain sections illustrating hemorrhagic foci in each group (left; scale bar, 200 μm), and quantification of hemorrhage area across groups (right; control, n = 6; 2-DG and 2-DG + CD8 Treg, n = 4).*

(G) *Representative immunofluorescence images of CD31 and ZO-1 staining in brain sections from mice receiving indicated treatments (left; scale bar, 100 μm), and quantification of ZO-1^{+} endothelial coverage across groups (right; n = 5 per group).*

(H) *Flow cytometry plots showing proportions of PD-L1^{+} neutrophils in different groups (left) and quantification of differences (right; control, n = 6; CD122Ab and CD8 Treg groups, n = 5).*

(I) *Representative bone marrow sections stained for Ly6G and PD-L1 (left), and quantification of PD-L1^{+} neutrophils (right; n = 5 per group; scale bar, 100 μm).*

(J–K) *Neurological recovery of PD-L1 blockade and control mice after MCAO, assessed by the adhesive removal test (J) and rotarod test (K) (Control n = 8; PD-L1Ab n = 7).*

(L) *Schematic illustration of the experimental workflow for PD-L1 block treatment in MCAO mice.*

(M) *Quantification of peripheral neutrophil counts in control, PD-L1 blockade, and PD-L1 blockade + CD8 Treg groups (n = 5 per group).*

(N) *Quantification of the proportion of hypersegmented neutrophils across groups (n = 5 per group).*

(O) *Representative HE-stained coronal brain sections illustrating hemorrhagic foci in each group (left; scale bar, 200 μm), and quantification of hemorrhage area across*

groups (right; control, n = 6; PD-L1 blockade and PD-L1 blockade + CD8 Treg, n = 4).

(P) *Representative immunofluorescence images of CD31 and ZO-1 staining in brain sections from mice receiving indicated treatments (left; scale bar, 100 μm), and quantification of ZO-1⁺ endothelial coverage across groups (right; n = 5 per group).*

*Data are presented as mean ± SD. NS, not significant; *p < 0.05, **p < 0.01, ***p < 0.001. Student's t test (I), one-way ANOVA (D-H, M-P), or two-way ANOVA (A, B, J, K) with Tukey's test.*

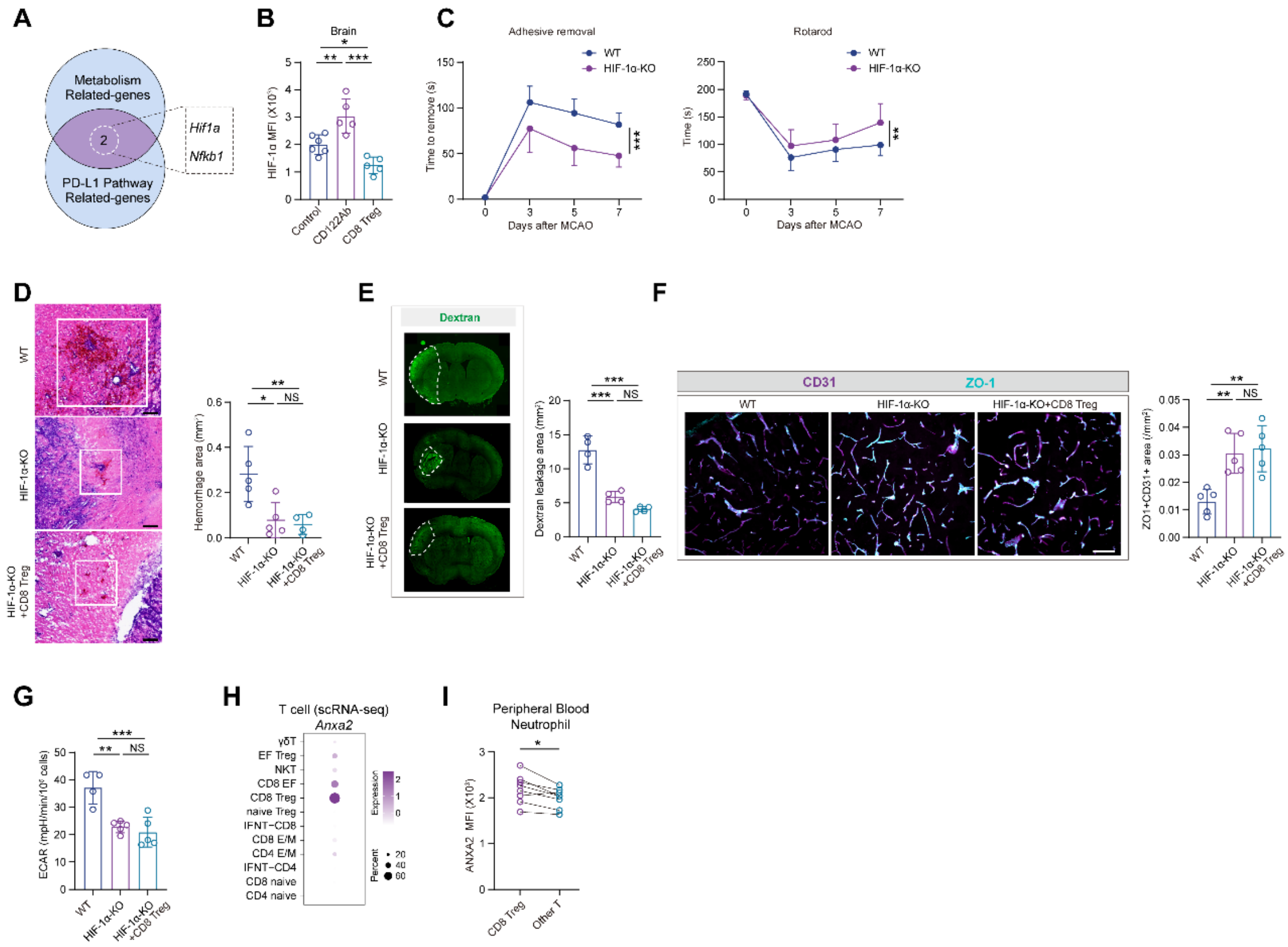

Figure S10. Role of HIF-1α in CD8 Treg–mediated regulation, related to Figure 6.

(A) *Intersection of metabolism-related and PD-L1–related pathways identifies Hif1a and Nfkb1 as overlapping genes.*

(B) *HIF-1α expression in brain-infiltrating neutrophils from control (n = 6), CD122Ab (n = 5), and CD8 Treg-treated mice (n = 5).*

(C) *Neurological recovery of HIF-1α-KO and WT mice after MCAO, assessed by the adhesive removal test and rotarod test (WT, n = 8; HIF-1α-KO, n = 7).*

(D) *Representative HE-stained coronal brain sections illustrating hemorrhagic foci in each group (left; scale bar, 200 μm), and quantification of hemorrhage area across groups (right; WT and HIF-1α-KO, n = 5; HIF-1α-KO + CD8 Treg, n = 4).*

(E) *Representative images of dextran leakage in brain sections (left) and leakage volume (right; n = 4 per group).*

(F) *Representative images of ZO-1 and CD31 staining in brain sections (left) and ZO-1^+ endothelial area across groups (right; n = 5 per group; scale bar, 100 μm).*

(G) *Glycolytic capacity of WT neutrophils (n = 4), HIF-1α–KO neutrophils (n = 5), and HIF-1α–KO neutrophils co-cultured with CD8 Tregs (n = 5).*

(H) *Dot plot showing Anxa2 expression across peripheral T cell clusters.*

(I) *Flow cytometry quantification of ANXA2 expression in CD8 Tregs and other T cell subsets (n = 8).*

*Data are presented as mean ± SD. NS, not significant; *p < 0.05, **p < 0.01, ***p < 0.001. Paired Student's t test (I), one-way ANOVA (B, D-G) or two-way ANOVA (C) with Tukey's test.*

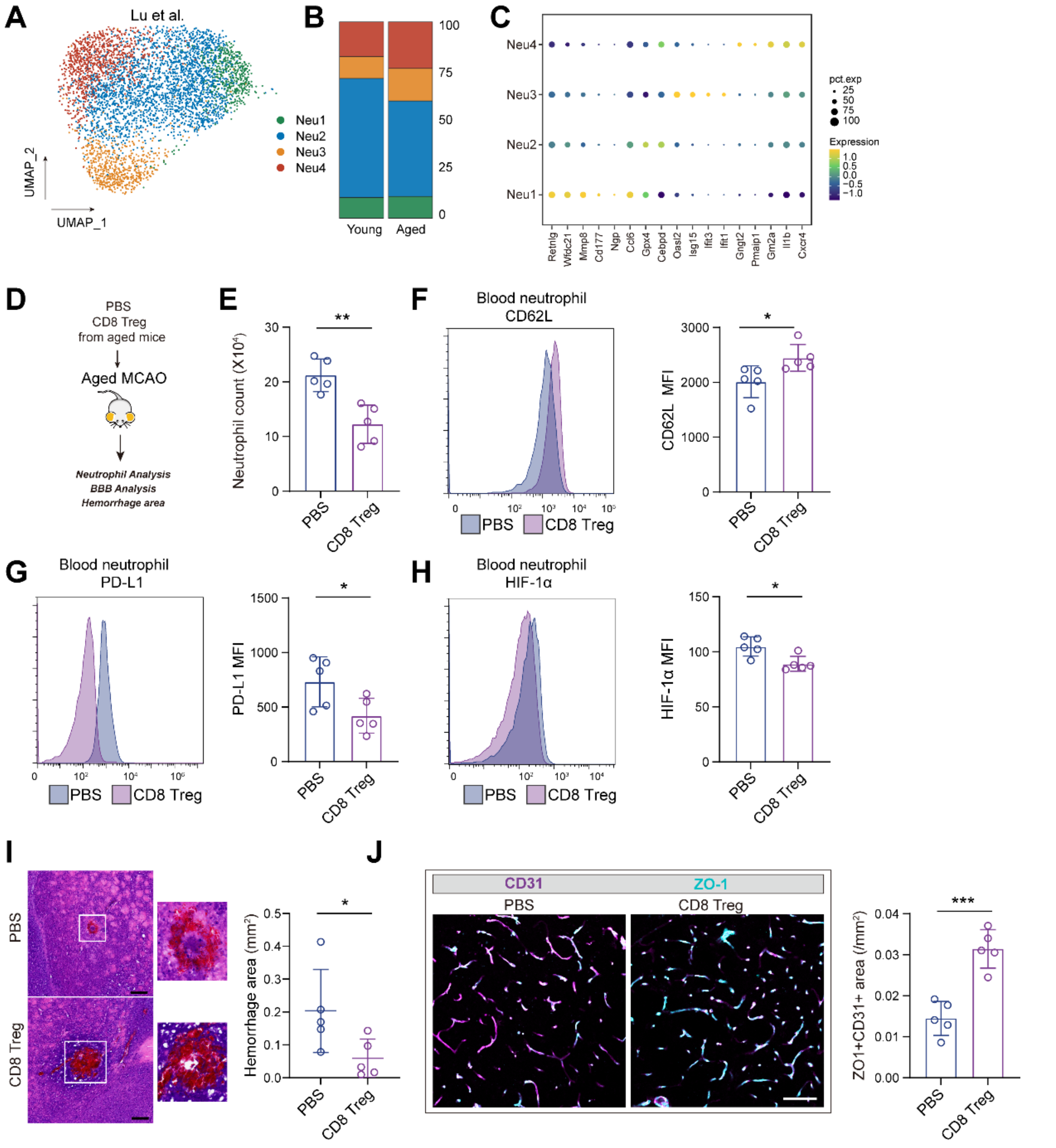

Figure S11. CD8 Treg–mediated regulation of neutrophil lifespan is preserved in aged mice.

(A) *UMAP visualization of peripheral neutrophil clusters from young and aged mice.*

(B) *Bar plot showing the proportional composition of neutrophil subclusters in young and aged mice.*

(C) *Dot plot showing the expression of canonical marker genes across neutrophil subclusters.*

(D) *Schematic illustration of the experimental workflow for adoptive transfer of CD8 Tregs into aged MCAO mice.*

(E) *Quantification of peripheral neutrophil counts in PBS and CD8 Treg transfer*

groups (n = 5 per group).

(F-H) *Comparison of CD62L MFI (F), PD-L1 MFI (G), and HIF-1α MFI (H) on peripheral neutrophils between PBS and CD8 Treg transfer groups (n = 5 per group).*

(I) *Representative HE-stained coronal brain sections illustrating hemorrhagic foci in each group (left; scale bar, 200 μm), and quantification of hemorrhage area across groups (right; n = 5 per group).*

(J) *Representative immunofluorescence images of CD31 and ZO-1 staining in brain sections from aged MCAO mice receiving indicated treatments (left; scale bar, 100 μm), and quantification of ZO-1^{+} endothelial coverage across groups (right; n = 5 per group).*

Data are presented as mean ± SD. $p < 0.05$ considered significant; Student's t test (E-J).

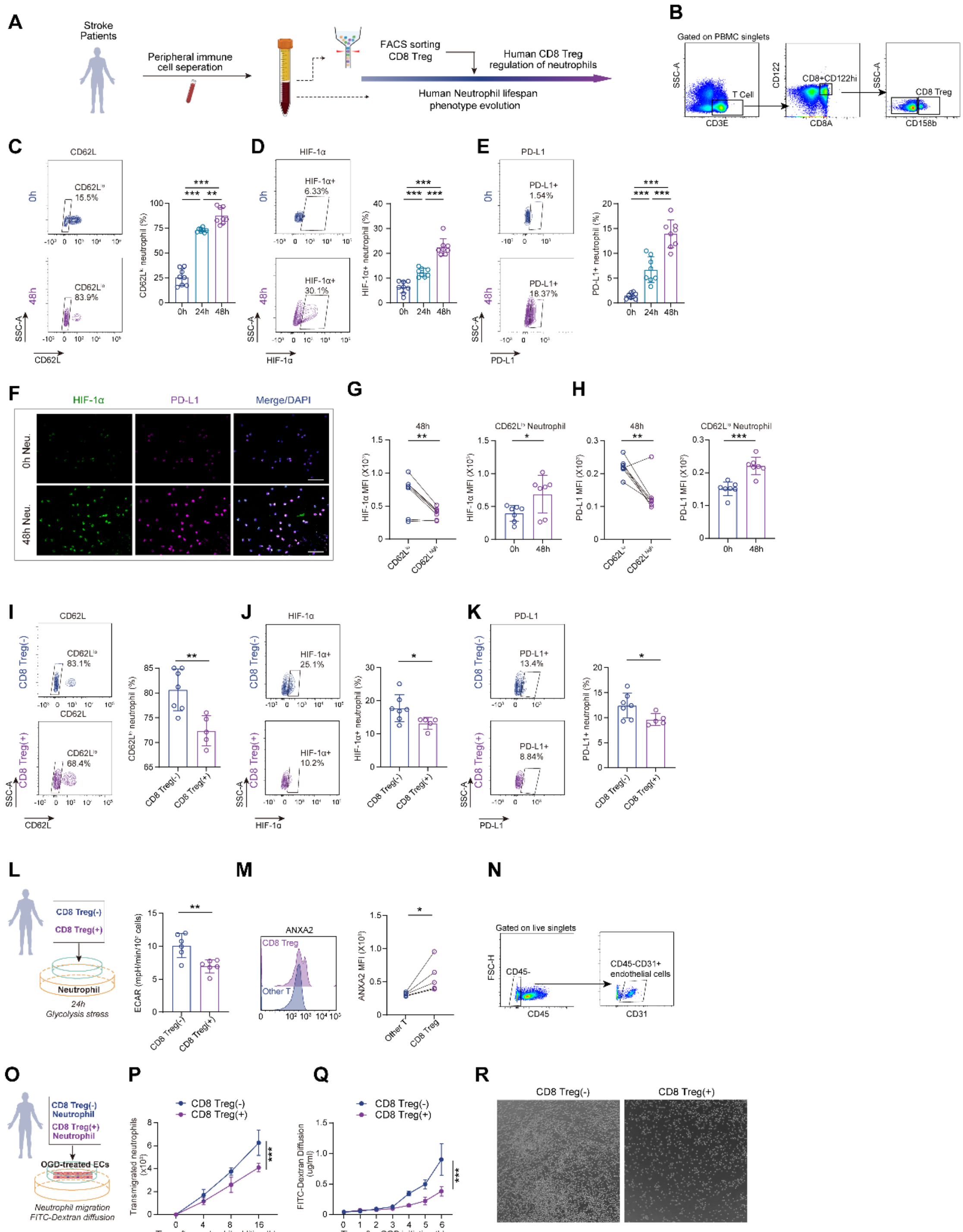

Figure S12. Human CD8 Tregs regulate neutrophil lifespan via mechanisms analogous to those observed in mice.

(A) *Schematic illustration of experimental workflow for cell isolation from stroke patients and subsequent assays.*

(B) *Gating strategy for flow cytometric identification of human CD8 Tregs.*

(C–E) *Flow cytometry plots showing expression of CD62L (C), HIF-1α (D), and PD-*

L1 (E) in neutrophils at different time points, with quantification of differences (n = 8 per group).

(F) *Immunofluorescence images showing HIF-1α and PD-L1 staining in human neutrophils (scale bar, 50 μm).*

(G) *Comparison of HIF-1α expression between $CD62L^{lo}$ and $CD62L^{hi}$ neutrophils at 48 h (left), and comparison of HIF-1α expression in $CD62L^{lo}$ neutrophils between 0 h and 48 h culture (right; n = 7 per group).*

(H) *Comparison of PD-L1 expression between $CD62L^{lo}$ and $CD62L^{hi}$ neutrophils at 48 h (left), and comparison of PD-L1 expression in $CD62L^{lo}$ neutrophils between 0 h and 48 h culture (right; n = 7 per group).*

(I–K) *Flow cytometry plots showing expression of CD62L (I), HIF-1α (J), and PD-L1 (K) in neutrophils under single-culture or co-culture conditions with CD8 Tregs, with group-wise quantification (neutrophils in single culture, n = 7, co-culture, n = 5).*

(L) *Schematic illustration of glycolytic function assays in neutrophils under single-culture or co-culture conditions with CD8 Tregs (left), and comparison of glycolytic capacity (right; n = 6 per group).*

(M) *Representative flow cytometry plots showing ANXA2 expression in CD8 Tregs and other T cell subsets from human peripheral blood (left), and quantification of ANXA2 expression between populations (right; n = 5).*

(N) *Gating strategy for isolation of endothelial cells from human brain tissue.*

(O) *Schematic illustration of neutrophil transendothelial migration and dextran permeability assays.*

(P) *Quantification of transmigrated neutrophil numbers at indicated timepoints (n = 4 per group). 0 h represents neutrophil addition.*

(Q) *Quantification of FITC–dextran leakage at indicated timepoints (n = 5 per group).*

(R) *Representative images of neutrophil transmigration assay under single-culture and CD8 Treg co-culture conditions.*

*Data are presented as mean ± SD. *p < 0.05, **p < 0.01, ***p < 0.001. Student's t test (G right, H right, I-L), paired Student's t test (G left, H left, M), one-way ANOVA (C-E), or two-way ANOVA (P, Q) with Tukey's test.*

Supplemental References

1. Kuhns DB, Priel DAL, Chu J, and Zarember KA. Isolation and Functional Analysis of Human Neutrophils. *Curr Protoc Immunol.* 2015;111:7 23 1-7 16.
2. Mao L, Li P, Zhu W, Cai W, Liu Z, Wang Y, et al. Regulatory T cells ameliorate tissue plasminogen activator-induced brain haemorrhage after stroke. *Brain.* 2017;140(7):1914-31.
3. Bucher K, Schmitt F, Autenrieth SE, Dillmann I, Nurnberg B, Schenke-Layland K, et al. Fluorescent Ly6G antibodies determine macrophage phagocytosis of neutrophils and alter the retrieval of neutrophils in mice. *J Leukoc Biol.* 2015;98(3):365-72.
4. Shi L, Sun Z, Su W, Xu F, Xie D, Zhang Q, et al. Treg cell-derived osteopontin promotes microglia-mediated white matter repair after ischemic stroke. *Immunity.* 2021;54(7):1527-42 e8.
5. Zhang Q, Zhu W, Xu F, Dai X, Shi L, Cai W, et al. The interleukin-4/PPARgamma signaling axis promotes oligodendrocyte differentiation and remyelination after brain injury. *PLoS Biol.* 2019;17(6):e3000330.
6. Shi Y, Zhang L, Pu H, Mao L, Hu X, Jiang X, et al. Rapid endothelial cytoskeletal reorganization enables early blood-brain barrier disruption and long-term ischaemic reperfusion brain injury. *Nat Commun.* 2016;7:10523.
7. Pang Z, Lu Y, Zhou G, Hui F, Xu L, Viau C, et al. MetaboAnalyst 6.0: towards a unified platform for metabolomics data processing, analysis and interpretation. *Nucleic Acids Res.* 2024;52(W1):W398-W406.
8. Jin S, Plikus MV, and Nie Q. CellChat for systematic analysis of cell-cell communication from single-cell transcriptomics. *Nat Protoc.* 2025;20(1):180-219.
9. Li Q. scTour: a deep learning architecture for robust inference and accurate prediction of cellular dynamics. *Genome Biol.* 2023;24(1):149.
10. Qiu X, Mao Q, Tang Y, Wang L, Chawla R, Pliner HA, et al. Reversed graph embedding resolves complex single-cell trajectories. *Nat Methods.* 2017;14(10):979-82.
11. Cao J, Spielmann M, Qiu X, Huang X, Ibrahim DM, Hill AJ, et al. The single-cell transcriptional landscape of mammalian organogenesis. *Nature.* 2019;566(7745):496-502.
12. Gulati GS, Sikandar SS, Wesche DJ, Manjunath A, Bharadwaj A, Berger MJ, et al. Single-cell transcriptional diversity is a hallmark of developmental potential. *Science.* 2020;367(6476):405-11.
